# Trends in incidence and correlation between medical costs and lost workdays for work‐related amputations in the State of California from 2007 to 2018

**DOI:** 10.1002/hsr2.319

**Published:** 2021-07-01

**Authors:** Nicholas G. Gomez, Fraser W. Gaspar, Matthew S. Thiese, Andrew S. Merryweather

**Affiliations:** ^1^ Department of Mechanical Engineering University of Utah Salt Lake City Utah USA; ^2^ MDGuidelines, ReedGroup Ltd Westminster Colorado USA; ^3^ Department of Family and Preventative Medicine – Rocky Mountain Center for Occupational and Environmental Health University of Utah Salt Lake City Utah USA

**Keywords:** amputations, electronic health records, long‐term disability, occupational health, prosthetics, public health, workers' compensation

## Abstract

**Background:**

Detailed information regarding workers who experience an amputation in the workplace over the last decade is limited. To better understand the financial and functional impact of a work‐related amputation, this study quantifies the incidence of work‐related amputations in the California workforce from 2007 to 2018 as well as the relationship between medical costs and lost workdays as a function of amputation level.

**Methods:**

Workers' compensation claims data from California spanning the years 2007 to 2018 were evaluated to describe trends in amputation incidence (N = 16 931). Quartile values for medical costs, indemnity costs, and lost workdays were reported as a function of amputation level. Correlations were performed between medical costs and lost workdays to examine their relationship.

**Results:**

The average incidence from 2007 to 2018 was 8.9 (95% CI 8.5, 9.4) amputations per 100 000 workers. There was a significant spike in amputations in 2008. Partial‐hand amputations were the most common with 73.3 (95% CI 69.2, 77.7) cases per 1 000 000 workers, and the industry with the highest incidence was construction with 26.0 (95% CI 22.4, 30.0) cases per 100 000 workers. Overall, medical costs were moderately correlated with lost workdays (Spearman's rho = 0.51), and that level of correlation remained relatively consistent across all levels of amputation (Spearman's rho = 0.48‐0.62).

**Conclusions:**

Amputations represent high medical costs and number of lost workdays. Considering the type of amputation and the industry the injury occurred in is important in order to work toward returning this population to work. Our results present the status of amputations in the California workplace and establish a basis for using medical costs to infer lost work productivity for this population.

## 1 INTRODUCTION

Each year, there are approximately 2.8 million nonfatal injuries reported in the United States.^1^ These injuries result in a national annual financial burden in excess of 186 billion dollars.^2^ In 2017, the National Council on Compensation Insurance identified that amputations represented the costliest injury managed by the workers' compensation system with almost double the costs compared to the next most expensive type of injury being fracture/crush/dislocation.^3^ Approximately one‐third of persons who experience an amputation in the workplace setting can never return to work, and those who do often have to change their occupation.^4^

Despite the disparity in costs for this type of injury, there is limited person‐level information available to help medical professionals and policymakers minimize the financial and functional impact associated with an amputation. To date, there have been a limited number of studies investigating the incidence of both upper and lower‐extremity work‐related amputations.^5^, ^6^, ^7^, ^8^, ^9^, ^10^ These studies vary in time intervals, ranging from a single year^7^, ^10^ to a 10‐year period.^5^, ^9^ The studies that investigated incidence rate over a long period of time identified rates ranging from approximately 13.7 to 38.8 claims per 100 000 workers, and manufacturing was the industry with the highest reported incidence.^5^, ^6^, ^9^ This coincides with the Occupational Safety and Health Administration's Directive Number CPL 03‐00‐019 enacted in 2015, which created a focus on the manufacturing industry in an effort to introduce engineering controls that reduce the risk of injuries resulting in an amputation.^11^

Though several authors have reported incidence estimates of amputations, the costs related to amputations are important to understand as medical costs have been associated with a loss of functional ability and, consequently, loss of workdays.^12^, ^13^, ^14^, ^15^ Total costs also represent the economic burden an amputation can have on both the injured worker and society. Little information has been published related to the costs associated with a work‐related amputation. McCall and Horwitz reported on indemnity costs according to amputation level and found that multiple upper and lower extremities carried the largest median indemnity costs ($25 729 and $27 000, respectively) and finger(s) were the most common with a median cost of $2113.^9^ Anderson et al reported average and median medical costs, indemnity costs, and lost workdays, but only for their whole study population and not stratified by amputation level.^6^ To date, indemnity costs, medical costs, and lost workdays have not been reported together by amputation level, which is necessary to provide a more complete picture of the impact of these catastrophic injuries. The relationship between costs and lost workdays has been enumerated for other health outcomes, such as musculoskeletal injuries, but not amputations.^12^, ^16^ Currently, it is unknown whether cost information for amputations provides similar indications of lost work productivity as it does for other injuries.^12^, ^13^, ^14^, ^15^ Depending on the strength of the correlation, knowing how medical costs and lost workdays correlate would allow for some conclusion regarding the lost work productivity to be made when only medical spending information is available. This correlation also elucidates the experience of the injured worker following their injury. A strong correlation would indicate that the worker receives medical care the entire time they are off work. A weak correlation would either indicate high medical costs for a short number of lost workdays or an extended period of time where the worker cannot work and is no longer receiving medical care.

Thus, this study aims to evaluate the incidence of amputations in the workplace setting and to understand the associations between the related medical costs and lost workdays. Specifically, an analysis of work‐related amputations was performed using workers' compensation claims data filed over a 12‐year period in California from 2007 to 2018. With a population of over 39 million, 63.1% of whom are in the civilian labor force, California has both the largest state population and largest state economy in the United States.^17^ The main objectives of this study are to (a) establish demographic information of workers who experienced a work‐related amputation, (b) quantitate annual incidence of amputations, (c) describe medical costs, indemnity costs, and lost workdays, and (d) determine the relationship between medical costs and lost workdays.

## 2 METHODS

### 2.1 Data source

This study used de‐identified claims data reported to the California Department of Industrial Relations (DIR) Workers' Compensation Information System (WCIS) from 1 January 2007 to 1 November 2019. The DIR granted the authors permission to use the claims data in the scope of this study without an Institutional Review Board review due to the de‐identified nature of the claims information. A data request was made to the WCIS requesting all information on claims that matched either a Healthcare Common Procedure Coding System (HCPCS) prosthetic base code or an ICD‐9/10‐CM diagnosis code or an ICD‐9/10‐CM/Current Procedural Terminology (CPT) procedure code related to an amputation. This request identified 25 555 claims, and the full details of the information available from WCIS can be found in their implementation guides.^18^, ^19^

### 2.2 Study population

The study population consisted of those who had a diagnosis or procedure code with enough specificity that could tie them to one of the following amputation levels: above‐elbow (forequarter, shoulder disarticulation, transhumeral, and elbow disarticulation), below‐elbow (transradial and wrist disarticulation), partial‐hand (transmetacarpal, finger(s), thumb), above‐knee (hip disarticulation, transfemoral, knee disarticulation), below‐knee (transtibial, Syme's), partial‐foot (midfoot, lesser toe(s), and great toe), or multiple (claim had identifiers for multiple levels, indicating scenarios such as a revision amputation or multiple amputations being present) (N = 24 345). The specific codes used to group claims into the aforementioned categories consisted of ICD‐9/10‐CM diagnosis codes, ICD‐9/10‐CM procedure codes, and CPT codes (Table S1). Of the claims that could be categorized (N = 24 345), we only included those that appeared on the subsequent report of injury (SROI) table (N = 17 622). We applied this restriction because the SROI table had all benefits information used to determine lost workdays and indemnity payments. Claims data from 2019 (N=691) were not complete regarding the ability to calculate complete demographic, proportion, and incidence results so we removed those claims making 16 931 the final number of claims identified for descriptive and incidence analyses. Since not all claims were listed as closed, only closed claims (N=11,699) were used to calculate medical costs, indemnity costs, lost workdays, and correlations.

### 2.3 Demographic information

For this study, both descriptive and derived data were pulled from the records. Worker age at the time of initial injury was calculated as the difference between the date of injury and date of birth in years. Worker gender was directly recorded in WCIS records in a binary fashion, either male or female. The industry was defined using 2‐digit North American Industry Classification Systems codes.^20^ The nature of injury was the type of injury that started the claim. It should be noted that the nature of injury that was reported to start the claim was determined by the subjective discretion of the individual filling out the first report of injury (FROI) form, which is why injuries outside of "amputation" are present. The presence of a prosthesis as part of the treatment protocol was determined by whether an appropriate HCPCS prosthetic base code was found in the medical records. Participation in a lawsuit was determined by a recorded date of representation. The annual salary was calculated using the weekly wage information as well as the payment frequency. When wage information was not available (N = 1669), it was imputed using the median salary reported for the worker's Standard Occupational Classification or North American Industry Classification Systems code.^20^, ^21^ The worker's employment status was directly recorded as full‐time, part‐time, or other. Medical complexity for a claim was defined as the number of unique medical visits and the number of unique medical diagnoses and procedure codes in the first month following the date of injury.

### 2.4 Calculation of incidence, medical costs, indemnity costs, and lost workdays

The incidence rate for the study population was calculated similarly to the published methodology.^5^ Annual claims were divided by the total number of workers reported by the Employment Development Department of the State of California (EDD) for the third quarter.^22^, ^23^ Just the third quarter was used because some data were archived and only had third quarter information available. Data from years with all four quarters were analyzed and there were no significant differences on a quarter‐to‐quarter basis. Amputation level‐specific incidence was calculated as the average of the annual events at each level divided by the average number of total workers reported by the EDD. The overall amputations and the level‐specific amputations were reported both on a yearly basis and as an annual average that averaged incidence over the 12‐year study period. Industry‐specific incidence was calculated as the average annual events reported for each industry in the claims data divided by the 12‐year average industry‐specific population of workers reported by the EDD.^22^, ^23^ Medical costs were calculated as the sum of medical, pharmaceutical, and durable medical equipment dollars paid over the duration of the closed claims. Lost workdays were calculated as the number of workdays a worker received temporary total disability benefits. Indemnity costs were defined as the summation of temporary total disability payments, permanent total/partial payments, employer‐paid payments, and lump‐sum payments. Permanent disability, employer‐paid benefits, and lump‐sum payments can cover both future medical costs and lost workdays.^24^ Those dollar amounts were defined as additional indemnity costs and reported separately as a percent of the total indemnity costs. All costs were reported as a function of amputation level and incorporated information from all years of the study population.

### 2.5 Statistical analysis

Claims data were processed and organized using SQLite.^25^ Trends in incidence were evaluated using a two‐sided chi‐square trend test according to total annual events as well as by amputation level. For the trend analysis, claims were only counted once and reported for the year the injury occurred, ensuring their independence. The relationship between medical costs and lost workdays was evaluated using Spearman's rank correlations and were stratified by amputation level. Trend and correlation analyses were performed in MATLAB^26^ using built‐in functionality and the Cochran‐Armitage function.^27^ Statistical significance was set at *P* < .05.

## 3 RESULTS

### 3.1 Demographics

Of the WCIS claims recorded from 2007 to 2018, 16 931 satisfied the inclusion criteria for this study. The median age was 42 years, and the age group with the largest representation was the 45 to 54 range (24.3%). This study population was comprised primarily of males (84.8%). The 50^th^ percentile salary from 2007 to 2018 was $31 200, with most of the claims falling in the $0‐24 999 category (34.3%). Most of the claimants were full‐time employees (71.7%). Only a small amount of the study population received prosthetic care (3.7%), and approximately one out of every five claims (19.2%) were documented to have legal action associated with them. In the first month following the documented date of injury, claims had a median of 9 unique medical visits and 22 unique diagnosis or procedure codes (Table 1). The majority of the claims consisted of partial‐hand amputations (82.0%). Of those classified as a person with a partial‐hand amputation, 66.7% were fingers without the loss of the thumb. Following partial‐hand amputations, partial‐foot amputations were the next most common at 5.9% of the claims (Table 2). The industry with the highest number of claims was manufacturing (24.1%). The most common nature of injury that started a claim that had an associated amputation as defined by our inclusion criteria (Table S1) was "Lacerations" (29.9%), followed closely by “Amputations” (26.7%) (Table 3).

**TABLE 1:** Demographics of persons who experienced a work‐related amputation in California from 2007 to 2018

	Summary values^d^
Average number of claims annually	1410.9
Average working population annually	15 783 315.7
Incidence (95% CI)^a^	8.9 (8.5‐9.4)
Age (y)^b^	30|**42**|52
16‐24^c^	160.5 (11.4%)
25‐34^c^	318.4 (22.6%)
35‐44^c^	318.8 (22.6%)
45‐54^c^	342.8 (24.3%)
55‐64^c^	225.0 (15.9%)
65+^c^	42.8 (3.0%)
Gender	
Male^c^	1197.1 (84.8%)
Female^c^	208.6 (14.8%)
Annual salary ($)^b^	21 630|**31 200**|47 482
0‐24 999^c^	483.5 (34.3%)
25 000‐34 999^c^	325.8 (23.1%)
35 000‐44 999^c^	211.1 (15.0%)
45 000‐54 999^c^	143.1 (10.1%)
55 000‐64 999^c^	81.1 (5.7%)
65 000‐74 000^c^	60.3 (4.3%)
75 000+^c^	104.6 (7.4%)
Prosthesis use^c^	51.6 (3.7%)
Presence of lawsuit^c^	270.8 (19.2%)
Employment status	
Full‐time^c^	1011.8 (71.7%)
Part‐time^c^	105.6 (7.5%)
Other^c^	292.8 (20.8%)
Medical complexity	
Medical visits^b^	5|**9**|13
Unique diagnoses^b^	13|**22**|33

^a^ per 100 000 workers.

^b^ 25^th^|**50** ^**th**^|75^th^ percentile values.

^c^ Average count (% of total).

^d^ Percentages may not add to 100 due to rounding.

**TABLE 2:** Average proportion and incidence of amputations stratified by amputation level

	‘07	‘08	‘09	‘10	‘11	‘12	‘13	‘14	‘15	‘16	‘17	‘18	Proportion of Total Claims (%)^c^	Average incidence per year (95%CI)^b^
Above‐Elbow	12	27	27	25	17	21	25	31	49	64	92	67	2.7	2.4 (1.7‐3.4)
Below‐Elbow	28	40	29	31	22	22	18	15	21	22	24	21	1.7	1.6 (1.0‐2.6)
Partial‐Hand	1224	2116	1332	1048	949	1019	902	1116	1102	870	1057	1154	82.0	73.3 (69.2‐77.7)
Just Finger^a^	921	1632	1102	869	787	840	754	952	889	730	878	941	66.7	59.6 (55.9‐63.6)
Just Thumb^a^	99	236	97	68	72	73	59	70	110	104	119	141	7.4	6.6 (5.4‐8.0)
Above‐Knee	11	25	18	14	15	19	12	15	15	16	18	12	1.1	1.0 (0.6‐1.7)
Below‐Knee	26	47	37	40	32	28	28	28	24	30	35	40	2.3	2.1 (1.5‐3.0)
Paritial‐Foot	76	117	101	73	77	85	68	81	82	78	82	72	5.9	5.2 (4.2‐6.5)
Multiple	48	92	68	61	67	45	43	40	64	62	63	62	4.2	3.8 (2.9‐4.9)
Total by Year	1425	2464	1612	1292	1179	1239	1096	1326	1357	1142	1371	1428		

^a^ Will not add to "Partial‐Hand" due to presence of other amputation levels in the "Partial‐Hand" group; Not included in "Total by Year" count.

^b^ per 1 000 000 workers.

^c^ Percentages possibly will not add to 100 due to rounding.

**TABLE 3:** Average annual cases and incidence stratified by industry and nature of injury from 2007 to 2018

Industry	Average	Average incidence per year^a^ ^,^ ^b^
Construction	186.7 (13.2%)	26.0 (22.4‐30.0)
Manufacturing	339.8 (24.1%)	25.9 (23.3‐28.9)
Retail Trade	132.3 (9.4%)	8.3 (6.9‐9.8)
Administrative and Support and Waste Management and Remediation Services	119.8 (8.5%)	12.0 (10.0‐14.4)
Agriculture, Forestry, Fishing, and Hunting	69.3 (4.9%)	15.1 (11.8‐19.2)
Wholesale Trade	60.1 (4.3%)	8.7 (6.7‐11.2)
Accommodation and Food Services	59.9 (4.2%)	4.2 (3.2‐5.4)
Transportation and Warehousing	54.4 (3.9%)	11.8 (9.0‐15.5)
Arts, Entertainment, and Recreation	41.3 (2.9%)	15.1 (11.0‐20.7)
Other Services (except Public Administration)	37.8 (2.7%)	6.0 (4.3‐8.3)
Professional, Scientific, and Technical Services	36.5 (2.6%)	3.2 (2.3‐4.5)
Public Administration	33.5 (2.4%)	1.4 (1.0‐2.0)
Other (<18%)^c^	239.7 (17.0%)	—
Nature of Injury		
Laceration	422.3 (29.9%)	26.8 (24.3‐29.5)
Amputation	377.1 (26.7%)	23.9 (21.6‐26.5)
Fracture	105.9 (7.5%)	6.7 (5.5‐8.2)
Crushing	105.8 (7.5%)	6.7 (5.5‐8.1)
Strain or Tear	93.1 (6.6%)	5.9 (4.8‐7.3)
All Other Specific Injuries, NOC	61.1 (4.3%)	3.9 (3.0‐5.0)
Severance	55.2 (3.9%)	3.5 (2.7‐4.6)
Contusion	48.7 (3.5%)	3.1 (2.3‐4.1)
Sprain or Tear	29.5 (2.1%)	1.9 (1.3‐2.7)
Multiple Physical Injuries Only	25.3 (1.8%)	1.6 (1.1‐2.4)
All Other Cumulative Injuries	21.9 (1.6%)	1.4 (0.9‐2.1)
Other (<5%)^c^	65 (4.6%)	—

^a^ Industry incidence per 100 000 workers and calculated with respect to 12‐year average worker population cited for those specific industries.^22^, ^23^

^b^ Nature of injury incidence per 1 000 000 workers and calculated with respect to total 12‐year average worker population.

^c^ contains claims with missing data.

### 3.2 Trends in incidence

**FIGURE 1:**
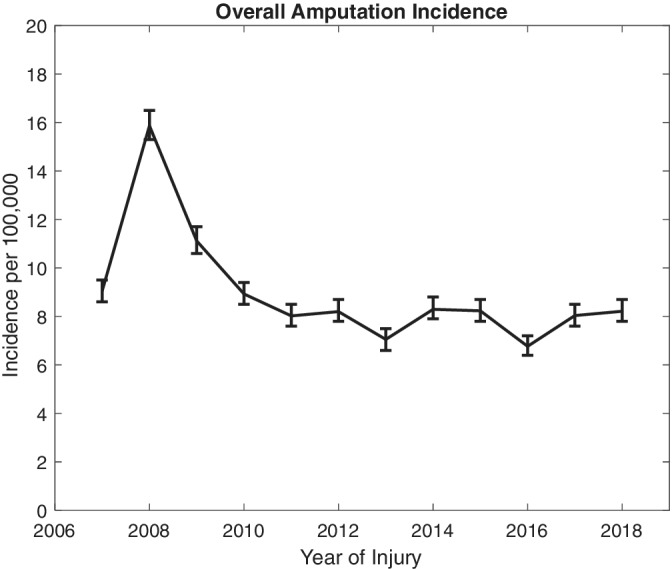
Overall annual incidence of amputations with 95% confidence intervals from 2007 to 2018

**FIGURE 2:**
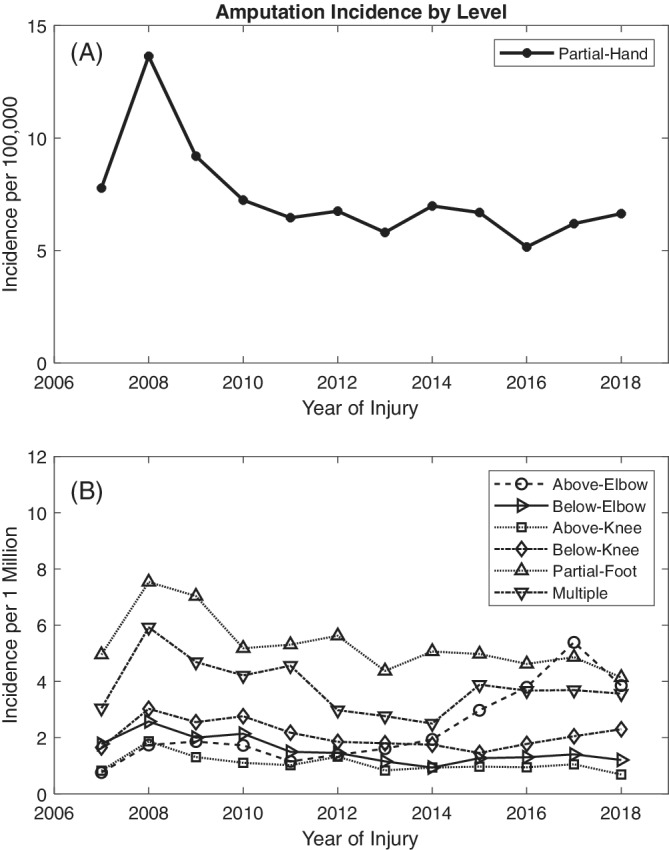
Annual incidence of amputations stratified by level from 2007 to 2018. A, Represents partial‐hand amputation incidence per 100 000 workers and B, represents above‐elbow, below‐elbow, above‐knee, below‐knee, partial‐foot, and multiple amputations per 1 000 000 workers

The average incidence over the 12‐year period under investigation was 8.9 (95%CI 8.5, 9.4) amputations per 100 000 workers (Table 1). Partial‐hand amputations had the highest overall incidence of 73.3 (95%CI 69.2, 77.7) cases per 1 000 000 workers (Table 2). The industry with the highest average incidence was construction at 26.0 (95% CI 22.4, 30.0) cases per 100 000 workers (Table 3; complete annual breakdown found in Table S2). There was a significant increase (*P* for trend <.001) in the total number of amputations from 2007 to 2008 (Figure 1). This was true for all amputation groups (*P* for trends <.05) except the below‐elbow group and was most noticeable in the partial‐hand group (Figure 2A). From 2009 to 2018, there was a decrease in overall incidence (*P* for trend <.001). Stratified by amputation level, the incidence of partial‐hand, below‐elbow, and partial‐foot amputations decreased (*P* for trends <.05), the incidence of above‐elbow amputations increased (*P* for trend <.001), and the incidence of above‐knee, below‐knee, and multiple amputations did not change significantly (*P* for trends >.05) (Figure 2B).

### 3.3 Summary of medical costs, indemnity costs, lost workdays, and correlations

In general, median medical costs, indemnity costs, and lost workdays increased with the severity of the amputation. Though partial‐hand amputations were the most common (Table 2), they had the lowest median medical costs ($7785), lowest median indemnity costs ($8279), and shortest median time off work (40 days) (Table 4). Despite the low median values, the sheer volume of partial‐hand amputations resulted in that group still having the highest proportion of medical costs (55.7%), indemnity costs (66.2%), and lost workdays (75.2%) compared to the other groups. The multiple amputation group accounted for 26.0% of the medical dollars paid toward claims associated with an amputation. Multiple amputations also had the highest median medical costs ($46 637), most median time off work (275 days), and the highest median indemnity costs ($54 583) (Table 4). Despite the general trend of an increase in costs and lost workdays with amputation severity, it should be noted that this increase did not hold true for the above‐elbow group compared to the below‐elbow group (Table 4). Also, the median values for total lost workdays and total indemnity costs were found to be comparable for the above‐knee and below‐knee groups (Table 4).

**TABLE 4:** Quartiles of medical costs, indemnity costs, and lost workdays and proportions of medical costs, indemnity costs, additional indemnity costs, and lost workdays from 2007 to 2018

	Medical costs ($)^a^	Medical costs proportion (%)^b^	Indemnity costs ($)^a^	Indemnity costs proportion (%)^b^	Additional indemnity cost proportion (%)^b^ ^,^ ^c^	Lost workdays (d)^a^	Proportion of lost workdays (%)^b^
Above‐Elbow	5073|**14 855**|29 129	1.4	5281|**17 302**|40 075	2.3	1.4	10|**80**|201	2.1
Below‐Elbow	8985|**30 825**|72 807	2.2	8192|**41 548**|111 410	3.9	2.8	16|**208**|507	2.8
Partial‐Hand	3254|**7785**|17 876	55.7	2550|**8279**|22 1136	66.2	39.8	9|**40**|128	75.2
Above‐Knee	5829|**18 390**|79 017	2.1	5955|**28 729**|75 452	2.3	1.8	7|**126**|518	1.6
Below‐Knee	6394|**20 901**|104 381	4.4	7975|**27 477**|81 762	5.9	4.8	4|**126**|470	3.3
Partial‐Foot	6252|**21 469**|59 592	8.3	7698|**21 406**|54 787	7.5	5.0	26|**105**|301	7.9
Multiple	13 379|**46 637**|155 020	26.0	16 342|**54 583**|128 401	12.0	9.3	56|**275**|551	7.2

^a^ 25^th^|**50** ^**th**^|75^th^ percentile values.

^b^ Percentages possibly will not add to 100 due to rounding.

^c^ Proportion of indemnity costs that do not include temporary total disability payments stratified by amputation level.

Medical costs and lost workdays showed an overall moderate‐to‐strong correlation (Spearman's rho = 0.48‐0.62) across amputation levels (Table 5). The strongest correlation was in the above‐knee group (Spearman's rho = 0.62), and the weakest correlation was the partial‐hand amputation group (Spearman's rho = 0.48) (Table 5).

**TABLE 5:** Spearman's correlation between medical costs and lost workdays

	Number of claims	Median medical costs ($)	Median lost workdays (d)	Spearman's Rho	*P*‐value
Above‐elbow	197	14 855	80	0.51	<.001
Below‐elbow	149	30 825	208	0.59	<.001
Partial‐hand	10 175	7785	40	0.48	<.001
Above‐knee	91	18 390	126	0.62	<.001
Below‐knee	194	20 901	126	0.57	<.001
Partial‐foot	592	21 469	105	0.50	<.001
Multiple	301	46 637	275	0.51	<.001
Total population	11 699	8590	42	0.51	<.001

## 4 DISCUSSION

Over the 12‐year time period being investigated in this study, we found that the 12‐year average total incidence was 8.9 (95%CI 8.5, 9.4) amputations per 100 000 workers. This value is comparable to previous studies investigating amputation incidence in the United States. Specifically, Michigan, Kentucky, Washington, and Minnesota reported 13.6, 13.7, 19.2, and 39 amputations per 100 000 workers, respectively.^6^, ^7^, ^8^, ^9^ Those same studies reported similar breakdowns as our study in terms of amputation type and industries in which they occurred. Manufacturing and construction were cited as the most common industries in which an amputation occurred, and finger amputations were the most common by a large margin. Our study agrees with those conclusions and indicates that the United States experienced on average less incidence than a country like Korea, which reported a national average annual incidence of 38.8 claims per 100 000 workers from 2004 to 2013.^5^ These differences are most likely the result of different safety standards as well as the nature of the specific jobs within each industry. However, despite the difference in incidence, the source of amputation risk, as well as the most common amputations are not nationally exclusive.

Our study found that in California, there was a substantial spike in incidence during 2008 where the rate almost doubled (Figure 1). This trend carried over into 2009 to some degree, but by 2010, the annual incidence rate returned to approximately the annual rate seen in 2007 and from 2010 to 2018. This spike correlates with the economic recession that the United States faced during that time period, in which California was one of the states most impacted.^28^ Some of the consequences of the recession included a retraction of the state and national economy as well as an increase in unemployment. When using annual employment numbers in this study to calculate incidence (Table S3), the comparison population saw the beginnings of a decline in 2008 (15.7‐15.5 million) followed by a large dip to 14.5 million in 2009. This drop follows the timing of the economic recession. The literature investigating the relationship between economic declines and worker health is inconclusive. Using global information since the recession was not exclusive to the United States, researchers in Spain concluded that the economic recession actually decreased the number of workplace injuries since only those who are most qualified tend to remain in the workforce when workforce contraction is imminent.^29^ Researchers in Italy also found a similar trend where the injury rates decreased starting in 2008.^30^ ^.^ However, other researchers concluded that the risk of some illnesses increases in the presence of economic decline. Still, individuals are able to adapt to the new social and economic conditions quickly.^31^ Our results support the idea that the recession potentially increased the risk of injury by making workers more susceptible to existing hazards in the workplace. The inherent hazards of the occupations did not change. Instead, worker response to external factors may have exacerbated the existing risks for amputations. This is supported by the high incidence of partial‐hand amputations in 2008 as well as the high incidence in the construction and manufacturing industries (Table 3). Both of those industries rely heavily on powered machines, which carry a high risk of causing an amputation when used in error.^32^ This concept challenges the safety measures currently in place, especially those for jobs where fingers are at risk because they rely heavily on worker compliance to prevent injury.^8^, ^11^ The interaction between the great recession and occupational injury events should be explored in future studies to determine if an increase in amputations was observed in other states or if that response was unique to the state of California.

Post 2008, there was a decrease in the incidence of amputations (*P* for trend <.001). It should be noted that 2009 showed a higher incidence compared to the average seen between 2010 and 2018, indicating that the effects of the recession were still present in terms of how it impacted the inherent risk of injury. The studies looking at incidence over time^5^, ^6^, ^9^ all reported a general decrease in the number of amputations which aligns with the findings of this study. In general, workplaces have progressively become safer over time. Looking at national, nonfatal occupation injury events from 2003 to 2018, the Bureau of Labor Statistics cites that the number of total recordable cases has decreased from 5.0 to 2.8 per 100 workers.^33^ In this case, the results for California indicate that apart from factors such as the potential effects seen from the economic recession, worker safety as it relates to risk for amputation has effectively decreased slightly over the last decade.

Medical costs were overall moderately correlated with lost workdays (Spearman's rho = 0.51) which was comparable to prior research investigating similar correlations.^12^ This result indicates that some inferences can be made regarding lost work productivity just by investigating medical spending. This is important for working with claims data as medical costs are generally documented much more thoroughly than lost workdays. In our results, partial‐hand and partial‐foot had the lowest correlation between medical costs and lost workdays, indicating that for those groups, costs are not necessarily a good metric for establishing functional limitations. These two groups tended to have more lost workdays and lower median medical costs compared to the other amputation groups. This indicates that persons in these groups likely continued to lose workdays after reaching maximum medical improvement due to not being able to perform previous job duties. Ideally, prosthetic care should return some amount of function. Still, the lower correlation speaks to the need for improvements, particularly at the partial‐hand and partial‐foot levels, which were also the two most prevalent levels.

Indemnity costs are designed to compensate injured workers by paying them a portion of their wage as well as covering medical costs.^34^ For less severe injuries, the majority of the indemnity payments go toward replacing lost wages in the form of temporary total disability payments. Our study found that a large number of claims involving persons who experienced an amputation in the workplace also had permanent disability payments associated with them as well as lump‐sum payouts and employer‐paid benefits that can cover both future medical costs as well as lost earnings (N = 8282). These costs are not often enumerated and are the result of scheduled benefits that accompany the loss of body parts. Additional indemnity comprised more than half of the total indemnity costs spent per amputation level (Table 4). These dollars should be noted as they cover medical care and lost workdays not documented in this study, as their exact intent is not recorded in the claims data. However, we do know they are intended to replace both wages and medical costs outside of what is documented in the claims.^24^ What that means for our results is that both the reported medical dollars and lost workdays are an underestimation of the true costs and lost productivity that can come from an amputation that occurs in the workplace.

Overall, we found investigating amputations on a person‐level basis resulted in scope of impact larger than has been reported by the Bureau of Labor Statistics (BLS). The BLS recently reported an annual national incidence of 5.0 amputations per 100 000 workers and 31 median lost workdays per claim.^35^ The main reason for the discrepancy was our data source and how we classified amputations. BLS categorizes amputations using the annual Survey of Occupational Injuries and Illnesses (SOII).^36^ That data source is self‐reported nationally by over 230,000 employers. The nature of injury on the SOII questionnaire comes from the Occupational Safety and Health Administration (OSHA) 300 logs and is subjectively determined by the company completing it, similar to how the nature of injury is determined on the WCIS first report of injury form. We found from the WCIS records that using the initial injury to define amputations was unreliable. Instead, investigating appropriate diagnosis or procedure codes from the medical records resulted in many additional claims (N = 12 406) outside of those that listed "amputation" as the nature of injury that started the claim. Consequently, since BLS SOII and workers' compensation data have been shown to report similarly,^37^, ^38^ we suspect that the BLS data also underestimate the incidence and impact of amputations.

### 4.1 Study limitations

This study had several limitations that impact the generalizability of the results. WCIS only tracks medical billing from claims managed by claims adjusters processing more than 150 claims per year.^18^ The number of claims this restriction impacts are unknown but is expected to be relatively small. The dataset itself included changes in documentation methodology over time, which limited the amount of consistent data across the whole 12‐year period. In 2012, the United States switched claim documentation from HIPPA 4010 to 5010, the latter of which contained more data for each claim.^39^ In 2015, the United States switched from ICD‐9‐CM to ICD‐10‐CM diagnosis and procedure codes, which made the classification of amputation level for this study difficult.^39^ The amputation groups were limited in specificity to the highest level of detail that was recorded by ICD‐9‐CM and CPT codes. ICD‐10‐CM codes contain a considerable amount of extra detail. Still, following the transition to the new codes in 2015, it was seen in the records that there was not an immediate adoption, and there was a large increase in the number of unspecified codes. Finally, approximately 30% of the claims were listed as "open" on the SROI table, which limited the number of claims that could be used to calculate costs and lost workdays.

## 5 CONCLUSION

Our findings identified that socioeconomic upheaval, such as found in the United States starting approximately 2008, can have a profound impact on worker safety. We also found that amputations follow the associations reported in previous research, which found that medical costs are moderately correlated with lost workdays.^21^ From 2007 to 2018, partial‐hand amputations had the highest incidence rate and resulted in the highest proportion of medical costs, indemnity costs, and lost workdays. Those who experienced multiple amputations had the highest median medical costs, indemnity costs, and lost workdays. Since the lost workdays were relatively high for all amputation levels, our results indicate there are significant functional and/or psychosocial barriers that need to be overcome in order to help individuals who experience an amputation in the workplace return to work. Also, though the incidence of amputations in the workplace is relatively low, they result in costs and lost workdays far greater than what has been reported for other workplace injuries.^3^ There is a large discrepancy in both costs and lost work productivity between levels of amputation, indicating that investigating this population beyond the mere presence of amputation is vital to understand the full impact of this type of injury. Future work should focus on the factors that impact costs and productivity loss post‐amputation to identify areas that can mitigate the impact of these catastrophic injuries.

## CONFLICT OF INTEREST

The authors declare that they have no conflict of interest.

## AUTHOR CONTRIBUTIONS

Conceptualization: Nicholas G. Gomez, Andrew S. Merryweather

Data Curation: Nicholas G. Gomez, Fraser W. Gaspar

Formal Analysis: Nicholas G. Gomez, Fraser W. Gaspar

Funding Acquisition: Matthew S. Thiese, Andrew S. Merryweather

Methodology: Nicholas G. Gomez, Fraser W. Gaspar, Matthew S. Thiese, Andrew S. Merryweather

Project Administration: Nicholas G. Gomez, Matthew S. Thiese, Andrew S. Merryweather

Supervision: Matthew S. Thiese, Andrew S. Merryweather

Writing‐Original Draft: Nicholas G. Gomez

Writing‐review and editing: Fraser W. Gaspar, Matthew S. Thiese, Andrew S. Merryweather

All authors have read and approved the final version of the manuscript.

Nicholas G. Gomez had full access to all of the data in this study and takes complete responsibility for the integrity of the data and the accuracy of the data analysis.

## TRANSPARENCY STATEMENT

Nicholas G. Gomez affirms that this manuscript is an honest, accurate, and transparent account of the study being reported; that no important aspects of the study have been omitted; and that any discrepancies from the study as planned (and, if relevant, registered) have been explained.

## Supporting information


**Table S1.** Codes used to define study population and each amputation group.

**Table S2.** Annual proportion and average incidence of amputations according to industry and nature of injury from 2007 to 2018.

**Table S3.** Complete annual demographics of persons who experienced a work‐related amputation in California from 2007 to 2018.

## Data Availability

Data subject to third party restrictions. The data that support the findings of this study are available from the California Department of Industrial Relations Workers' Compensation Information System. Restrictions apply to the availability of these data, which were used under license for this study.
