# Supplementary material for: Trends in incidence and correlation between medical costs and lost workdays for work‐related amputations in the State of California from 2007 to 2018
Source: Health Sci Rep. 2021 Jul 1;4(3):e319. doi: 10.1002/hsr2.319 (PMC8247939; doi:10.1002/hsr2.319)
Supplement: Supplementary file 1 — Table S1. Codes used to define study population and each amputation group. [file HSR2-4-e319-s003.docx]

Supplementary 1: Codes used to define study population and each amputation group

| **Code** | **Code Type** | **Amputation group code was used to define inclusion criteria** |
| --- | --- | --- |
| 24920 | CPT | Above Elbow |
| 24925 | CPT | Above Elbow |
| 24930 | CPT | Above Elbow |
| 24931 | CPT | Above Elbow |
| 24935 | CPT | Above Elbow |
| 24940 | CPT | Above Elbow |
| 23900 | CPT | Above Elbow |
| 23920 | CPT | Above Elbow |
| 23921 | CPT | Above Elbow |
| 24900 | CPT | Above Elbow |
| 0X690Z2 | ICD 10 Procedure | Above Elbow |
| 0X690Z3 | ICD 10 Procedure | Above Elbow |
| 0X6C0ZZ | ICD 10 Procedure | Above Elbow |
| 0X610ZZ | ICD 10 Procedure | Above Elbow |
| 0X630ZZ | ICD 10 Procedure | Above Elbow |
| 0X690Z1 | ICD 10 Procedure | Above Elbow |
| 0X680Z2 | ICD 10 Procedure | Above Elbow |
| 0X680Z3 | ICD 10 Procedure | Above Elbow |
| 0X6B0ZZ | ICD 10 Procedure | Above Elbow |
| 0X600ZZ | ICD 10 Procedure | Above Elbow |
| 0X620ZZ | ICD 10 Procedure | Above Elbow |
| 0X680Z1 | ICD 10 Procedure | Above Elbow |
| S48.012A | ICD 10 Diagnosis | Above Elbow |
| S48.012D | ICD 10 Diagnosis | Above Elbow |
| S48.012S | ICD 10 Diagnosis | Above Elbow |
| S48.022 | ICD 10 Diagnosis | Above Elbow |
| S48.022A | ICD 10 Diagnosis | Above Elbow |
| S48.022D | ICD 10 Diagnosis | Above Elbow |
| S48.022S | ICD 10 Diagnosis | Above Elbow |
| S48.112 | ICD 10 Diagnosis | Above Elbow |
| S48.112A | ICD 10 Diagnosis | Above Elbow |
| S48.112D | ICD 10 Diagnosis | Above Elbow |
| S48.112S | ICD 10 Diagnosis | Above Elbow |
| S48.122 | ICD 10 Diagnosis | Above Elbow |
| S48.122A | ICD 10 Diagnosis | Above Elbow |
| S48.122D | ICD 10 Diagnosis | Above Elbow |
| S48.122S | ICD 10 Diagnosis | Above Elbow |
| S48.912 | ICD 10 Diagnosis | Above Elbow |
| S48.912A | ICD 10 Diagnosis | Above Elbow |
| S48.912D | ICD 10 Diagnosis | Above Elbow |
| S48.912S | ICD 10 Diagnosis | Above Elbow |
| S48.922 | ICD 10 Diagnosis | Above Elbow |
| S48.922A | ICD 10 Diagnosis | Above Elbow |
| S48.922D | ICD 10 Diagnosis | Above Elbow |
| S48.922S | ICD 10 Diagnosis | Above Elbow |
| S58.012 | ICD 10 Diagnosis | Above Elbow |
| S58.012A | ICD 10 Diagnosis | Above Elbow |
| S58.012D | ICD 10 Diagnosis | Above Elbow |
| S58.012S | ICD 10 Diagnosis | Above Elbow |
| S58.022 | ICD 10 Diagnosis | Above Elbow |
| S58.022A | ICD 10 Diagnosis | Above Elbow |
| S58.022D | ICD 10 Diagnosis | Above Elbow |
| S58.022S | ICD 10 Diagnosis | Above Elbow |
| Z89.222 | ICD 10 Diagnosis | Above Elbow |
| Z89.232 | ICD 10 Diagnosis | Above Elbow |
| S48.012 | ICD 10 Diagnosis | Above Elbow |
| S48.011A | ICD 10 Diagnosis | Above Elbow |
| S48.011D | ICD 10 Diagnosis | Above Elbow |
| S48.011S | ICD 10 Diagnosis | Above Elbow |
| S48.021 | ICD 10 Diagnosis | Above Elbow |
| S48.021A | ICD 10 Diagnosis | Above Elbow |
| S48.021D | ICD 10 Diagnosis | Above Elbow |
| S48.021S | ICD 10 Diagnosis | Above Elbow |
| S48.111 | ICD 10 Diagnosis | Above Elbow |
| S48.111A | ICD 10 Diagnosis | Above Elbow |
| S48.111D | ICD 10 Diagnosis | Above Elbow |
| S48.111S | ICD 10 Diagnosis | Above Elbow |
| S48.121 | ICD 10 Diagnosis | Above Elbow |
| S48.121A | ICD 10 Diagnosis | Above Elbow |
| S48.121D | ICD 10 Diagnosis | Above Elbow |
| S48.121S | ICD 10 Diagnosis | Above Elbow |
| S48.911 | ICD 10 Diagnosis | Above Elbow |
| S48.911A | ICD 10 Diagnosis | Above Elbow |
| S48.911D | ICD 10 Diagnosis | Above Elbow |
| S48.911S | ICD 10 Diagnosis | Above Elbow |
| S48.921 | ICD 10 Diagnosis | Above Elbow |
| S48.921A | ICD 10 Diagnosis | Above Elbow |
| S48.921D | ICD 10 Diagnosis | Above Elbow |
| S48.921S | ICD 10 Diagnosis | Above Elbow |
| S58.011 | ICD 10 Diagnosis | Above Elbow |
| S58.011A | ICD 10 Diagnosis | Above Elbow |
| S58.011D | ICD 10 Diagnosis | Above Elbow |
| S58.011S | ICD 10 Diagnosis | Above Elbow |
| S58.021 | ICD 10 Diagnosis | Above Elbow |
| S58.021A | ICD 10 Diagnosis | Above Elbow |
| S58.021D | ICD 10 Diagnosis | Above Elbow |
| S58.021S | ICD 10 Diagnosis | Above Elbow |
| Z89.221 | ICD 10 Diagnosis | Above Elbow |
| Z89.231 | ICD 10 Diagnosis | Above Elbow |
| S48.011 | ICD 10 Diagnosis | Above Elbow |
| S48.0 | ICD 10 Diagnosis | Above Elbow |
| S48.01 | ICD 10 Diagnosis | Above Elbow |
| S48.019 | ICD 10 Diagnosis | Above Elbow |
| S48.019A | ICD 10 Diagnosis | Above Elbow |
| S48.019D | ICD 10 Diagnosis | Above Elbow |
| S48.019S | ICD 10 Diagnosis | Above Elbow |
| S48.02 | ICD 10 Diagnosis | Above Elbow |
| S48.029 | ICD 10 Diagnosis | Above Elbow |
| S48.029A | ICD 10 Diagnosis | Above Elbow |
| S48.029D | ICD 10 Diagnosis | Above Elbow |
| S48.029S | ICD 10 Diagnosis | Above Elbow |
| S48.1 | ICD 10 Diagnosis | Above Elbow |
| S48.11 | ICD 10 Diagnosis | Above Elbow |
| S48.119 | ICD 10 Diagnosis | Above Elbow |
| S48.119A | ICD 10 Diagnosis | Above Elbow |
| S48.119D | ICD 10 Diagnosis | Above Elbow |
| S48.119S | ICD 10 Diagnosis | Above Elbow |
| S48.12 | ICD 10 Diagnosis | Above Elbow |
| S48.129 | ICD 10 Diagnosis | Above Elbow |
| S48.129A | ICD 10 Diagnosis | Above Elbow |
| S48.129D | ICD 10 Diagnosis | Above Elbow |
| S48.129S | ICD 10 Diagnosis | Above Elbow |
| S48.9 | ICD 10 Diagnosis | Above Elbow |
| S48.91 | ICD 10 Diagnosis | Above Elbow |
| S48.919 | ICD 10 Diagnosis | Above Elbow |
| S48.919A | ICD 10 Diagnosis | Above Elbow |
| S48.919D | ICD 10 Diagnosis | Above Elbow |
| S48.919S | ICD 10 Diagnosis | Above Elbow |
| S48.92 | ICD 10 Diagnosis | Above Elbow |
| S48.929 | ICD 10 Diagnosis | Above Elbow |
| S48.929A | ICD 10 Diagnosis | Above Elbow |
| S48.929D | ICD 10 Diagnosis | Above Elbow |
| S48.929S | ICD 10 Diagnosis | Above Elbow |
| S58 | ICD 10 Diagnosis | Above Elbow |
| S58.0 | ICD 10 Diagnosis | Above Elbow |
| S58.01 | ICD 10 Diagnosis | Above Elbow |
| S58.019 | ICD 10 Diagnosis | Above Elbow |
| S58.019A | ICD 10 Diagnosis | Above Elbow |
| S58.019D | ICD 10 Diagnosis | Above Elbow |
| S58.019S | ICD 10 Diagnosis | Above Elbow |
| S58.02 | ICD 10 Diagnosis | Above Elbow |
| S58.029 | ICD 10 Diagnosis | Above Elbow |
| S58.029A | ICD 10 Diagnosis | Above Elbow |
| S58.029D | ICD 10 Diagnosis | Above Elbow |
| S58.029S | ICD 10 Diagnosis | Above Elbow |
| Z89.229 | ICD 10 Diagnosis | Above Elbow |
| Z89.239 | ICD 10 Diagnosis | Above Elbow |
| S48 | ICD 10 Diagnosis | Above Elbow |
| 84.06 | ICD 9 Procedure | Above Elbow |
| 84.08 | ICD 9 Procedure | Above Elbow |
| 84.09 | ICD 9 Procedure | Above Elbow |
| 84.07 | ICD 9 Procedure | Above Elbow |
| 887.3 | ICD 9 Diagnosis | Above Elbow |
| 887.2 | ICD 9 Diagnosis | Above Elbow |
| 25905 | CPT | Below Elbow |
| 25907 | CPT | Below Elbow |
| 25909 | CPT | Below Elbow |
| 25915 | CPT | Below Elbow |
| 25920 | CPT | Below Elbow |
| 25922 | CPT | Below Elbow |
| 25924 | CPT | Below Elbow |
| 25927 | CPT | Below Elbow |
| 25929 | CPT | Below Elbow |
| 25931 | CPT | Below Elbow |
| 25900 | CPT | Below Elbow |
| 0X6F0Z2 | ICD 10 Procedure | Below Elbow |
| 0X6F0Z3 | ICD 10 Procedure | Below Elbow |
| 0X6K0Z0 | ICD 10 Procedure | Below Elbow |
| 0X6F0Z1 | ICD 10 Procedure | Below Elbow |
| 0X6D0Z2 | ICD 10 Procedure | Below Elbow |
| 0X6D0Z3 | ICD 10 Procedure | Below Elbow |
| 0X6J0Z0 | ICD 10 Procedure | Below Elbow |
| 0X6D0Z1 | ICD 10 Procedure | Below Elbow |
| S58.112A | ICD 10 Diagnosis | Below Elbow |
| S58.112D | ICD 10 Diagnosis | Below Elbow |
| S58.112S | ICD 10 Diagnosis | Below Elbow |
| S58.122 | ICD 10 Diagnosis | Below Elbow |
| S58.122A | ICD 10 Diagnosis | Below Elbow |
| S58.122D | ICD 10 Diagnosis | Below Elbow |
| S58.122S | ICD 10 Diagnosis | Below Elbow |
| S58.912 | ICD 10 Diagnosis | Below Elbow |
| S58.912A | ICD 10 Diagnosis | Below Elbow |
| S58.912D | ICD 10 Diagnosis | Below Elbow |
| S58.912S | ICD 10 Diagnosis | Below Elbow |
| S58.922 | ICD 10 Diagnosis | Below Elbow |
| S58.922A | ICD 10 Diagnosis | Below Elbow |
| S58.922D | ICD 10 Diagnosis | Below Elbow |
| S58.922S | ICD 10 Diagnosis | Below Elbow |
| S68.412 | ICD 10 Diagnosis | Below Elbow |
| S68.412A | ICD 10 Diagnosis | Below Elbow |
| S68.412D | ICD 10 Diagnosis | Below Elbow |
| S68.412S | ICD 10 Diagnosis | Below Elbow |
| S68.422 | ICD 10 Diagnosis | Below Elbow |
| S68.422A | ICD 10 Diagnosis | Below Elbow |
| S68.422D | ICD 10 Diagnosis | Below Elbow |
| S68.422S | ICD 10 Diagnosis | Below Elbow |
| Z89.112 | ICD 10 Diagnosis | Below Elbow |
| Z89.122 | ICD 10 Diagnosis | Below Elbow |
| Z89.212 | ICD 10 Diagnosis | Below Elbow |
| S58.112 | ICD 10 Diagnosis | Below Elbow |
| S58.111A | ICD 10 Diagnosis | Below Elbow |
| S58.111D | ICD 10 Diagnosis | Below Elbow |
| S58.111S | ICD 10 Diagnosis | Below Elbow |
| S58.121 | ICD 10 Diagnosis | Below Elbow |
| S58.121A | ICD 10 Diagnosis | Below Elbow |
| S58.121D | ICD 10 Diagnosis | Below Elbow |
| S58.121S | ICD 10 Diagnosis | Below Elbow |
| S58.911 | ICD 10 Diagnosis | Below Elbow |
| S58.911A | ICD 10 Diagnosis | Below Elbow |
| S58.911D | ICD 10 Diagnosis | Below Elbow |
| S58.911S | ICD 10 Diagnosis | Below Elbow |
| S58.921 | ICD 10 Diagnosis | Below Elbow |
| S58.921A | ICD 10 Diagnosis | Below Elbow |
| S58.921D | ICD 10 Diagnosis | Below Elbow |
| S58.921S | ICD 10 Diagnosis | Below Elbow |
| S68.411 | ICD 10 Diagnosis | Below Elbow |
| S68.411A | ICD 10 Diagnosis | Below Elbow |
| S68.411D | ICD 10 Diagnosis | Below Elbow |
| S68.411S | ICD 10 Diagnosis | Below Elbow |
| S68.421 | ICD 10 Diagnosis | Below Elbow |
| S68.421A | ICD 10 Diagnosis | Below Elbow |
| S68.421D | ICD 10 Diagnosis | Below Elbow |
| S68.421S | ICD 10 Diagnosis | Below Elbow |
| Z89.111 | ICD 10 Diagnosis | Below Elbow |
| Z89.121 | ICD 10 Diagnosis | Below Elbow |
| Z89.211 | ICD 10 Diagnosis | Below Elbow |
| S58.111 | ICD 10 Diagnosis | Below Elbow |
| S58.11 | ICD 10 Diagnosis | Below Elbow |
| S58.119 | ICD 10 Diagnosis | Below Elbow |
| S58.119A | ICD 10 Diagnosis | Below Elbow |
| S58.119D | ICD 10 Diagnosis | Below Elbow |
| S58.119S | ICD 10 Diagnosis | Below Elbow |
| S58.12 | ICD 10 Diagnosis | Below Elbow |
| S58.129 | ICD 10 Diagnosis | Below Elbow |
| S58.129A | ICD 10 Diagnosis | Below Elbow |
| S58.129D | ICD 10 Diagnosis | Below Elbow |
| S58.129S | ICD 10 Diagnosis | Below Elbow |
| S58.9 | ICD 10 Diagnosis | Below Elbow |
| S58.91 | ICD 10 Diagnosis | Below Elbow |
| S58.919 | ICD 10 Diagnosis | Below Elbow |
| S58.919A | ICD 10 Diagnosis | Below Elbow |
| S58.919D | ICD 10 Diagnosis | Below Elbow |
| S58.919S | ICD 10 Diagnosis | Below Elbow |
| S58.92 | ICD 10 Diagnosis | Below Elbow |
| S58.929 | ICD 10 Diagnosis | Below Elbow |
| S58.929A | ICD 10 Diagnosis | Below Elbow |
| S58.929D | ICD 10 Diagnosis | Below Elbow |
| S58.929S | ICD 10 Diagnosis | Below Elbow |
| S68 | ICD 10 Diagnosis | Below Elbow |
| S68.4 | ICD 10 Diagnosis | Below Elbow |
| S68.41 | ICD 10 Diagnosis | Below Elbow |
| S68.419 | ICD 10 Diagnosis | Below Elbow |
| S68.419A | ICD 10 Diagnosis | Below Elbow |
| S68.419D | ICD 10 Diagnosis | Below Elbow |
| S68.419S | ICD 10 Diagnosis | Below Elbow |
| S68.42 | ICD 10 Diagnosis | Below Elbow |
| S68.429 | ICD 10 Diagnosis | Below Elbow |
| S68.429A | ICD 10 Diagnosis | Below Elbow |
| S68.429D | ICD 10 Diagnosis | Below Elbow |
| S68.429S | ICD 10 Diagnosis | Below Elbow |
| Z89.1 | ICD 10 Diagnosis | Below Elbow |
| Z89.11 | ICD 10 Diagnosis | Below Elbow |
| Z89.119 | ICD 10 Diagnosis | Below Elbow |
| Z89.12 | ICD 10 Diagnosis | Below Elbow |
| Z89.129 | ICD 10 Diagnosis | Below Elbow |
| Z89.219 | ICD 10 Diagnosis | Below Elbow |
| S58.1 | ICD 10 Diagnosis | Below Elbow |
| 84.04 | ICD 9 Procedure | Below Elbow |
| 84.05 | ICD 9 Procedure | Below Elbow |
| 887.1 | ICD 9 Diagnosis | Below Elbow |
| 887.0 | ICD 9 Diagnosis | Below Elbow |
| 26951 | CPT | Partial Hand |
| 26952 | CPT | Partial Hand |
| 26910 | CPT | Partial Hand |
| 0X6K0Z5 | ICD 10 Procedure | Partial Hand |
| 0X6K0Z6 | ICD 10 Procedure | Partial Hand |
| 0X6K0Z7 | ICD 10 Procedure | Partial Hand |
| 0X6K0Z8 | ICD 10 Procedure | Partial Hand |
| 0X6K0Z9 | ICD 10 Procedure | Partial Hand |
| 0X6K0ZB | ICD 10 Procedure | Partial Hand |
| 0X6K0ZC | ICD 10 Procedure | Partial Hand |
| 0X6K0ZD | ICD 10 Procedure | Partial Hand |
| 0X6K0ZF | ICD 10 Procedure | Partial Hand |
| 0X6P0Z0 | ICD 10 Procedure | Partial Hand |
| 0X6P0Z1 | ICD 10 Procedure | Partial Hand |
| 0X6P0Z2 | ICD 10 Procedure | Partial Hand |
| 0X6P0Z3 | ICD 10 Procedure | Partial Hand |
| 0X6R0Z0 | ICD 10 Procedure | Partial Hand |
| 0X6R0Z1 | ICD 10 Procedure | Partial Hand |
| 0X6R0Z2 | ICD 10 Procedure | Partial Hand |
| 0X6R0Z3 | ICD 10 Procedure | Partial Hand |
| 0X6T0Z0 | ICD 10 Procedure | Partial Hand |
| 0X6T0Z1 | ICD 10 Procedure | Partial Hand |
| 0X6T0Z2 | ICD 10 Procedure | Partial Hand |
| 0X6T0Z3 | ICD 10 Procedure | Partial Hand |
| 0X6W0Z0 | ICD 10 Procedure | Partial Hand |
| 0X6W0Z1 | ICD 10 Procedure | Partial Hand |
| 0X6W0Z2 | ICD 10 Procedure | Partial Hand |
| 0X6W0Z3 | ICD 10 Procedure | Partial Hand |
| 0X6K0Z4 | ICD 10 Procedure | Partial Hand |
| 0X6J0Z5 | ICD 10 Procedure | Partial Hand |
| 0X6J0Z6 | ICD 10 Procedure | Partial Hand |
| 0X6J0Z7 | ICD 10 Procedure | Partial Hand |
| 0X6J0Z8 | ICD 10 Procedure | Partial Hand |
| 0X6J0Z9 | ICD 10 Procedure | Partial Hand |
| 0X6J0ZB | ICD 10 Procedure | Partial Hand |
| 0X6J0ZC | ICD 10 Procedure | Partial Hand |
| 0X6J0ZD | ICD 10 Procedure | Partial Hand |
| 0X6J0ZF | ICD 10 Procedure | Partial Hand |
| 0X6N0Z0 | ICD 10 Procedure | Partial Hand |
| 0X6N0Z1 | ICD 10 Procedure | Partial Hand |
| 0X6N0Z2 | ICD 10 Procedure | Partial Hand |
| 0X6N0Z3 | ICD 10 Procedure | Partial Hand |
| 0X6Q0Z0 | ICD 10 Procedure | Partial Hand |
| 0X6Q0Z1 | ICD 10 Procedure | Partial Hand |
| 0X6Q0Z2 | ICD 10 Procedure | Partial Hand |
| 0X6Q0Z3 | ICD 10 Procedure | Partial Hand |
| 0X6S0Z0 | ICD 10 Procedure | Partial Hand |
| 0X6S0Z1 | ICD 10 Procedure | Partial Hand |
| 0X6S0Z2 | ICD 10 Procedure | Partial Hand |
| 0X6S0Z3 | ICD 10 Procedure | Partial Hand |
| 0X6V0Z0 | ICD 10 Procedure | Partial Hand |
| 0X6V0Z1 | ICD 10 Procedure | Partial Hand |
| 0X6V0Z2 | ICD 10 Procedure | Partial Hand |
| 0X6V0Z3 | ICD 10 Procedure | Partial Hand |
| 0X6J0Z5 | ICD 10 Procedure | Partial Hand |
| 0X6M0Z1 | ICD 10 Procedure | Partial Hand |
| 0X6M0Z2 | ICD 10 Procedure | Partial Hand |
| 0X6M0Z3 | ICD 10 Procedure | Partial Hand |
| 0X6M0Z0 | ICD 10 Procedure | Partial Hand |
| 0X6L0Z1 | ICD 10 Procedure | Partial Hand |
| 0X6L0Z2 | ICD 10 Procedure | Partial Hand |
| 0X6L0Z3 | ICD 10 Procedure | Partial Hand |
| 0X6L0Z0 | ICD 10 Procedure | Partial Hand |
| S68.111A | ICD 10 Diagnosis | Partial Hand |
| S68.111D | ICD 10 Diagnosis | Partial Hand |
| S68.111S | ICD 10 Diagnosis | Partial Hand |
| S68.113 | ICD 10 Diagnosis | Partial Hand |
| S68.113A | ICD 10 Diagnosis | Partial Hand |
| S68.113D | ICD 10 Diagnosis | Partial Hand |
| S68.113S | ICD 10 Diagnosis | Partial Hand |
| S68.115 | ICD 10 Diagnosis | Partial Hand |
| S68.115A | ICD 10 Diagnosis | Partial Hand |
| S68.115D | ICD 10 Diagnosis | Partial Hand |
| S68.115S | ICD 10 Diagnosis | Partial Hand |
| S68.117 | ICD 10 Diagnosis | Partial Hand |
| S68.117A | ICD 10 Diagnosis | Partial Hand |
| S68.117D | ICD 10 Diagnosis | Partial Hand |
| S68.117S | ICD 10 Diagnosis | Partial Hand |
| S68.121 | ICD 10 Diagnosis | Partial Hand |
| S68.121A | ICD 10 Diagnosis | Partial Hand |
| S68.121D | ICD 10 Diagnosis | Partial Hand |
| S68.121S | ICD 10 Diagnosis | Partial Hand |
| S68.123 | ICD 10 Diagnosis | Partial Hand |
| S68.123A | ICD 10 Diagnosis | Partial Hand |
| S68.123D | ICD 10 Diagnosis | Partial Hand |
| S68.123S | ICD 10 Diagnosis | Partial Hand |
| S68.125 | ICD 10 Diagnosis | Partial Hand |
| S68.125A | ICD 10 Diagnosis | Partial Hand |
| S68.125D | ICD 10 Diagnosis | Partial Hand |
| S68.125S | ICD 10 Diagnosis | Partial Hand |
| S68.127 | ICD 10 Diagnosis | Partial Hand |
| S68.127A | ICD 10 Diagnosis | Partial Hand |
| S68.127D | ICD 10 Diagnosis | Partial Hand |
| S68.127S | ICD 10 Diagnosis | Partial Hand |
| S68.611 | ICD 10 Diagnosis | Partial Hand |
| S68.611A | ICD 10 Diagnosis | Partial Hand |
| S68.611D | ICD 10 Diagnosis | Partial Hand |
| S68.611S | ICD 10 Diagnosis | Partial Hand |
| S68.613 | ICD 10 Diagnosis | Partial Hand |
| S68.613A | ICD 10 Diagnosis | Partial Hand |
| S68.613D | ICD 10 Diagnosis | Partial Hand |
| S68.613S | ICD 10 Diagnosis | Partial Hand |
| S68.615 | ICD 10 Diagnosis | Partial Hand |
| S68.615A | ICD 10 Diagnosis | Partial Hand |
| S68.615D | ICD 10 Diagnosis | Partial Hand |
| S68.615S | ICD 10 Diagnosis | Partial Hand |
| S68.617 | ICD 10 Diagnosis | Partial Hand |
| S68.617A | ICD 10 Diagnosis | Partial Hand |
| S68.617D | ICD 10 Diagnosis | Partial Hand |
| S68.617S | ICD 10 Diagnosis | Partial Hand |
| S68.621 | ICD 10 Diagnosis | Partial Hand |
| S68.621A | ICD 10 Diagnosis | Partial Hand |
| S68.621D | ICD 10 Diagnosis | Partial Hand |
| S68.621S | ICD 10 Diagnosis | Partial Hand |
| S68.623 | ICD 10 Diagnosis | Partial Hand |
| S68.623A | ICD 10 Diagnosis | Partial Hand |
| S68.623D | ICD 10 Diagnosis | Partial Hand |
| S68.623S | ICD 10 Diagnosis | Partial Hand |
| S68.625 | ICD 10 Diagnosis | Partial Hand |
| S68.625A | ICD 10 Diagnosis | Partial Hand |
| S68.625D | ICD 10 Diagnosis | Partial Hand |
| S68.625S | ICD 10 Diagnosis | Partial Hand |
| S68.627 | ICD 10 Diagnosis | Partial Hand |
| S68.627A | ICD 10 Diagnosis | Partial Hand |
| S68.627D | ICD 10 Diagnosis | Partial Hand |
| S68.627S | ICD 10 Diagnosis | Partial Hand |
| S68.712 | ICD 10 Diagnosis | Partial Hand |
| S68.712A | ICD 10 Diagnosis | Partial Hand |
| S68.712D | ICD 10 Diagnosis | Partial Hand |
| S68.712S | ICD 10 Diagnosis | Partial Hand |
| S68.722 | ICD 10 Diagnosis | Partial Hand |
| S68.722A | ICD 10 Diagnosis | Partial Hand |
| S68.722D | ICD 10 Diagnosis | Partial Hand |
| S68.722S | ICD 10 Diagnosis | Partial Hand |
| Z89.022 | ICD 10 Diagnosis | Partial Hand |
| S68.111 | ICD 10 Diagnosis | Partial Hand |
| S68.110A | ICD 10 Diagnosis | Partial Hand |
| S68.110D | ICD 10 Diagnosis | Partial Hand |
| S68.110S | ICD 10 Diagnosis | Partial Hand |
| S68.112 | ICD 10 Diagnosis | Partial Hand |
| S68.112A | ICD 10 Diagnosis | Partial Hand |
| S68.112D | ICD 10 Diagnosis | Partial Hand |
| S68.112S | ICD 10 Diagnosis | Partial Hand |
| S68.114 | ICD 10 Diagnosis | Partial Hand |
| S68.114A | ICD 10 Diagnosis | Partial Hand |
| S68.114D | ICD 10 Diagnosis | Partial Hand |
| S68.114S | ICD 10 Diagnosis | Partial Hand |
| S68.116 | ICD 10 Diagnosis | Partial Hand |
| S68.116A | ICD 10 Diagnosis | Partial Hand |
| S68.116D | ICD 10 Diagnosis | Partial Hand |
| S68.116S | ICD 10 Diagnosis | Partial Hand |
| S68.120 | ICD 10 Diagnosis | Partial Hand |
| S68.120A | ICD 10 Diagnosis | Partial Hand |
| S68.120D | ICD 10 Diagnosis | Partial Hand |
| S68.120S | ICD 10 Diagnosis | Partial Hand |
| S68.122 | ICD 10 Diagnosis | Partial Hand |
| S68.122A | ICD 10 Diagnosis | Partial Hand |
| S68.122D | ICD 10 Diagnosis | Partial Hand |
| S68.122S | ICD 10 Diagnosis | Partial Hand |
| S68.124 | ICD 10 Diagnosis | Partial Hand |
| S68.124A | ICD 10 Diagnosis | Partial Hand |
| S68.124D | ICD 10 Diagnosis | Partial Hand |
| S68.124S | ICD 10 Diagnosis | Partial Hand |
| S68.126 | ICD 10 Diagnosis | Partial Hand |
| S68.126A | ICD 10 Diagnosis | Partial Hand |
| S68.126D | ICD 10 Diagnosis | Partial Hand |
| S68.126S | ICD 10 Diagnosis | Partial Hand |
| S68.610 | ICD 10 Diagnosis | Partial Hand |
| S68.610A | ICD 10 Diagnosis | Partial Hand |
| S68.610D | ICD 10 Diagnosis | Partial Hand |
| S68.610S | ICD 10 Diagnosis | Partial Hand |
| S68.612 | ICD 10 Diagnosis | Partial Hand |
| S68.612A | ICD 10 Diagnosis | Partial Hand |
| S68.612D | ICD 10 Diagnosis | Partial Hand |
| S68.612S | ICD 10 Diagnosis | Partial Hand |
| S68.614 | ICD 10 Diagnosis | Partial Hand |
| S68.614A | ICD 10 Diagnosis | Partial Hand |
| S68.614D | ICD 10 Diagnosis | Partial Hand |
| S68.614S | ICD 10 Diagnosis | Partial Hand |
| S68.616 | ICD 10 Diagnosis | Partial Hand |
| S68.616A | ICD 10 Diagnosis | Partial Hand |
| S68.616D | ICD 10 Diagnosis | Partial Hand |
| S68.616S | ICD 10 Diagnosis | Partial Hand |
| S68.620 | ICD 10 Diagnosis | Partial Hand |
| S68.620A | ICD 10 Diagnosis | Partial Hand |
| S68.620D | ICD 10 Diagnosis | Partial Hand |
| S68.620S | ICD 10 Diagnosis | Partial Hand |
| S68.622 | ICD 10 Diagnosis | Partial Hand |
| S68.622A | ICD 10 Diagnosis | Partial Hand |
| S68.622D | ICD 10 Diagnosis | Partial Hand |
| S68.622S | ICD 10 Diagnosis | Partial Hand |
| S68.624 | ICD 10 Diagnosis | Partial Hand |
| S68.624A | ICD 10 Diagnosis | Partial Hand |
| S68.624D | ICD 10 Diagnosis | Partial Hand |
| S68.624S | ICD 10 Diagnosis | Partial Hand |
| S68.626 | ICD 10 Diagnosis | Partial Hand |
| S68.626A | ICD 10 Diagnosis | Partial Hand |
| S68.626D | ICD 10 Diagnosis | Partial Hand |
| S68.626S | ICD 10 Diagnosis | Partial Hand |
| S68.711 | ICD 10 Diagnosis | Partial Hand |
| S68.711A | ICD 10 Diagnosis | Partial Hand |
| S68.711D | ICD 10 Diagnosis | Partial Hand |
| S68.711S | ICD 10 Diagnosis | Partial Hand |
| S68.721 | ICD 10 Diagnosis | Partial Hand |
| S68.721A | ICD 10 Diagnosis | Partial Hand |
| S68.721D | ICD 10 Diagnosis | Partial Hand |
| S68.721S | ICD 10 Diagnosis | Partial Hand |
| Z89.021 | ICD 10 Diagnosis | Partial Hand |
| S68.110 | ICD 10 Diagnosis | Partial Hand |
| S68.11 | ICD 10 Diagnosis | Partial Hand |
| S68.118 | ICD 10 Diagnosis | Partial Hand |
| S68.118A | ICD 10 Diagnosis | Partial Hand |
| S68.118D | ICD 10 Diagnosis | Partial Hand |
| S68.118S | ICD 10 Diagnosis | Partial Hand |
| S68.12 | ICD 10 Diagnosis | Partial Hand |
| S68.128 | ICD 10 Diagnosis | Partial Hand |
| S68.128A | ICD 10 Diagnosis | Partial Hand |
| S68.128D | ICD 10 Diagnosis | Partial Hand |
| S68.128S | ICD 10 Diagnosis | Partial Hand |
| S68.6 | ICD 10 Diagnosis | Partial Hand |
| S68.61 | ICD 10 Diagnosis | Partial Hand |
| S68.618 | ICD 10 Diagnosis | Partial Hand |
| S68.618A | ICD 10 Diagnosis | Partial Hand |
| S68.618D | ICD 10 Diagnosis | Partial Hand |
| S68.618S | ICD 10 Diagnosis | Partial Hand |
| S68.619 | ICD 10 Diagnosis | Partial Hand |
| S68.619A | ICD 10 Diagnosis | Partial Hand |
| S68.619D | ICD 10 Diagnosis | Partial Hand |
| S68.619S | ICD 10 Diagnosis | Partial Hand |
| S68.62 | ICD 10 Diagnosis | Partial Hand |
| S68.628 | ICD 10 Diagnosis | Partial Hand |
| S68.628A | ICD 10 Diagnosis | Partial Hand |
| S68.628D | ICD 10 Diagnosis | Partial Hand |
| S68.628S | ICD 10 Diagnosis | Partial Hand |
| S68.629 | ICD 10 Diagnosis | Partial Hand |
| S68.629A | ICD 10 Diagnosis | Partial Hand |
| S68.629D | ICD 10 Diagnosis | Partial Hand |
| S68.629S | ICD 10 Diagnosis | Partial Hand |
| S68.7 | ICD 10 Diagnosis | Partial Hand |
| S68.71 | ICD 10 Diagnosis | Partial Hand |
| S68.719 | ICD 10 Diagnosis | Partial Hand |
| S68.719A | ICD 10 Diagnosis | Partial Hand |
| S68.719D | ICD 10 Diagnosis | Partial Hand |
| S68.719S | ICD 10 Diagnosis | Partial Hand |
| S68.72 | ICD 10 Diagnosis | Partial Hand |
| S68.729 | ICD 10 Diagnosis | Partial Hand |
| S68.729A | ICD 10 Diagnosis | Partial Hand |
| S68.729D | ICD 10 Diagnosis | Partial Hand |
| S68.729S | ICD 10 Diagnosis | Partial Hand |
| Z89.0 | ICD 10 Diagnosis | Partial Hand |
| Z89.02 | ICD 10 Diagnosis | Partial Hand |
| Z89.029 | ICD 10 Diagnosis | Partial Hand |
| S68.1 | ICD 10 Diagnosis | Partial Hand |
| S68.012A | ICD 10 Diagnosis | Partial Hand |
| S68.012D | ICD 10 Diagnosis | Partial Hand |
| S68.012S | ICD 10 Diagnosis | Partial Hand |
| S68.022 | ICD 10 Diagnosis | Partial Hand |
| S68.022A | ICD 10 Diagnosis | Partial Hand |
| S68.022D | ICD 10 Diagnosis | Partial Hand |
| S68.022S | ICD 10 Diagnosis | Partial Hand |
| S68.512 | ICD 10 Diagnosis | Partial Hand |
| S68.512A | ICD 10 Diagnosis | Partial Hand |
| S68.512D | ICD 10 Diagnosis | Partial Hand |
| S68.512S | ICD 10 Diagnosis | Partial Hand |
| S68.522 | ICD 10 Diagnosis | Partial Hand |
| S68.522A | ICD 10 Diagnosis | Partial Hand |
| S68.522D | ICD 10 Diagnosis | Partial Hand |
| S68.522S | ICD 10 Diagnosis | Partial Hand |
| Z89.012 | ICD 10 Diagnosis | Partial Hand |
| S68.012 | ICD 10 Diagnosis | Partial Hand |
| S68.011A | ICD 10 Diagnosis | Partial Hand |
| S68.011D | ICD 10 Diagnosis | Partial Hand |
| S68.011S | ICD 10 Diagnosis | Partial Hand |
| S68.021 | ICD 10 Diagnosis | Partial Hand |
| S68.021A | ICD 10 Diagnosis | Partial Hand |
| S68.021D | ICD 10 Diagnosis | Partial Hand |
| S68.021S | ICD 10 Diagnosis | Partial Hand |
| S68.511 | ICD 10 Diagnosis | Partial Hand |
| S68.511A | ICD 10 Diagnosis | Partial Hand |
| S68.511D | ICD 10 Diagnosis | Partial Hand |
| S68.511S | ICD 10 Diagnosis | Partial Hand |
| S68.521 | ICD 10 Diagnosis | Partial Hand |
| S68.521A | ICD 10 Diagnosis | Partial Hand |
| S68.521D | ICD 10 Diagnosis | Partial Hand |
| S68.521S | ICD 10 Diagnosis | Partial Hand |
| Z89.011 | ICD 10 Diagnosis | Partial Hand |
| S68.011 | ICD 10 Diagnosis | Partial Hand |
| S68.01 | ICD 10 Diagnosis | Partial Hand |
| S68.019 | ICD 10 Diagnosis | Partial Hand |
| S68.019A | ICD 10 Diagnosis | Partial Hand |
| S68.019D | ICD 10 Diagnosis | Partial Hand |
| S68.019S | ICD 10 Diagnosis | Partial Hand |
| S68.02 | ICD 10 Diagnosis | Partial Hand |
| S68.029 | ICD 10 Diagnosis | Partial Hand |
| S68.029A | ICD 10 Diagnosis | Partial Hand |
| S68.029D | ICD 10 Diagnosis | Partial Hand |
| S68.029S | ICD 10 Diagnosis | Partial Hand |
| S68.5 | ICD 10 Diagnosis | Partial Hand |
| S68.51 | ICD 10 Diagnosis | Partial Hand |
| S68.519 | ICD 10 Diagnosis | Partial Hand |
| S68.519A | ICD 10 Diagnosis | Partial Hand |
| S68.519D | ICD 10 Diagnosis | Partial Hand |
| S68.519S | ICD 10 Diagnosis | Partial Hand |
| S68.52 | ICD 10 Diagnosis | Partial Hand |
| S68.529 | ICD 10 Diagnosis | Partial Hand |
| S68.529A | ICD 10 Diagnosis | Partial Hand |
| S68.529D | ICD 10 Diagnosis | Partial Hand |
| S68.529S | ICD 10 Diagnosis | Partial Hand |
| Z89.01 | ICD 10 Diagnosis | Partial Hand |
| Z89.019 | ICD 10 Diagnosis | Partial Hand |
| S68.0 | ICD 10 Diagnosis | Partial Hand |
| 84.03 | ICD 9 Procedure | Partial Hand |
| 84.01 | ICD 9 Procedure | Partial Hand |
| 84.02 | ICD 9 Procedure | Partial Hand |
| 886.1 | ICD 9 Diagnosis | Partial Hand |
| 886.0 | ICD 9 Diagnosis | Partial Hand |
| 885.1 | ICD 9 Diagnosis | Partial Hand |
| 885.0 | ICD 9 Diagnosis | Partial Hand |
| 27290 | CPT | Above Knee |
| 27295 | CPT | Above Knee |
| 27590 | CPT | Above Knee |
| 27591 | CPT | Above Knee |
| 27592 | CPT | Above Knee |
| 27594 | CPT | Above Knee |
| 27596 | CPT | Above Knee |
| 27598 | CPT | Above Knee |
| 0Y6G0ZZ | ICD 10 Procedure | Above Knee |
| 0Y6D0Z1 | ICD 10 Procedure | Above Knee |
| 0Y6D0Z2 | ICD 10 Procedure | Above Knee |
| 0Y6D0Z3 | ICD 10 Procedure | Above Knee |
| 0Y680ZZ | ICD 10 Procedure | Above Knee |
| 0Y630ZZ | ICD 10 Procedure | Above Knee |
| 0Y6F0ZZ | ICD 10 Procedure | Above Knee |
| 0Y6C0Z1 | ICD 10 Procedure | Above Knee |
| 0Y6C0Z2 | ICD 10 Procedure | Above Knee |
| 0Y6C0Z3 | ICD 10 Procedure | Above Knee |
| 0Y670ZZ | ICD 10 Procedure | Above Knee |
| 0Y620ZZ | ICD 10 Procedure | Above Knee |
| S88.012 | ICD 10 Diagnosis | Above Knee |
| S88.012A | ICD 10 Diagnosis | Above Knee |
| S88.012D | ICD 10 Diagnosis | Above Knee |
| S88.012S | ICD 10 Diagnosis | Above Knee |
| S88.022 | ICD 10 Diagnosis | Above Knee |
| S88.022A | ICD 10 Diagnosis | Above Knee |
| S88.022D | ICD 10 Diagnosis | Above Knee |
| S88.022S | ICD 10 Diagnosis | Above Knee |
| S78.012 | ICD 10 Diagnosis | Above Knee |
| S78.012A | ICD 10 Diagnosis | Above Knee |
| S78.012D | ICD 10 Diagnosis | Above Knee |
| S78.012S | ICD 10 Diagnosis | Above Knee |
| S78.022 | ICD 10 Diagnosis | Above Knee |
| S78.022A | ICD 10 Diagnosis | Above Knee |
| S78.022D | ICD 10 Diagnosis | Above Knee |
| S78.022S | ICD 10 Diagnosis | Above Knee |
| S78.112 | ICD 10 Diagnosis | Above Knee |
| S78.112A | ICD 10 Diagnosis | Above Knee |
| S78.112D | ICD 10 Diagnosis | Above Knee |
| S78.112S | ICD 10 Diagnosis | Above Knee |
| S78.122 | ICD 10 Diagnosis | Above Knee |
| S78.122A | ICD 10 Diagnosis | Above Knee |
| S78.122D | ICD 10 Diagnosis | Above Knee |
| S78.122S | ICD 10 Diagnosis | Above Knee |
| S78.912 | ICD 10 Diagnosis | Above Knee |
| S78.912A | ICD 10 Diagnosis | Above Knee |
| S78.912D | ICD 10 Diagnosis | Above Knee |
| S78.912S | ICD 10 Diagnosis | Above Knee |
| S78.922 | ICD 10 Diagnosis | Above Knee |
| S78.922A | ICD 10 Diagnosis | Above Knee |
| S78.922D | ICD 10 Diagnosis | Above Knee |
| S78.922S | ICD 10 Diagnosis | Above Knee |
| Z89.522 | ICD 10 Diagnosis | Above Knee |
| Z89.612 | ICD 10 Diagnosis | Above Knee |
| Z89.622 | ICD 10 Diagnosis | Above Knee |
| S88.011 | ICD 10 Diagnosis | Above Knee |
| S88.011A | ICD 10 Diagnosis | Above Knee |
| S88.011D | ICD 10 Diagnosis | Above Knee |
| S88.011S | ICD 10 Diagnosis | Above Knee |
| S88.021 | ICD 10 Diagnosis | Above Knee |
| S88.021A | ICD 10 Diagnosis | Above Knee |
| S88.021D | ICD 10 Diagnosis | Above Knee |
| S88.021S | ICD 10 Diagnosis | Above Knee |
| S78.011 | ICD 10 Diagnosis | Above Knee |
| S78.011A | ICD 10 Diagnosis | Above Knee |
| S78.011D | ICD 10 Diagnosis | Above Knee |
| S78.011S | ICD 10 Diagnosis | Above Knee |
| S78.021 | ICD 10 Diagnosis | Above Knee |
| S78.021A | ICD 10 Diagnosis | Above Knee |
| S78.021D | ICD 10 Diagnosis | Above Knee |
| S78.021S | ICD 10 Diagnosis | Above Knee |
| S78.111 | ICD 10 Diagnosis | Above Knee |
| S78.111A | ICD 10 Diagnosis | Above Knee |
| S78.111D | ICD 10 Diagnosis | Above Knee |
| S78.111S | ICD 10 Diagnosis | Above Knee |
| S78.121 | ICD 10 Diagnosis | Above Knee |
| S78.121A | ICD 10 Diagnosis | Above Knee |
| S78.121D | ICD 10 Diagnosis | Above Knee |
| S78.121S | ICD 10 Diagnosis | Above Knee |
| S78.911 | ICD 10 Diagnosis | Above Knee |
| S78.911A | ICD 10 Diagnosis | Above Knee |
| S78.911D | ICD 10 Diagnosis | Above Knee |
| S78.911S | ICD 10 Diagnosis | Above Knee |
| S78.921 | ICD 10 Diagnosis | Above Knee |
| S78.921A | ICD 10 Diagnosis | Above Knee |
| S78.921D | ICD 10 Diagnosis | Above Knee |
| S78.921S | ICD 10 Diagnosis | Above Knee |
| Z89.521 | ICD 10 Diagnosis | Above Knee |
| Z89.611 | ICD 10 Diagnosis | Above Knee |
| Z89.621 | ICD 10 Diagnosis | Above Knee |
| S88.019 | ICD 10 Diagnosis | Above Knee |
| S88.019A | ICD 10 Diagnosis | Above Knee |
| S88.019D | ICD 10 Diagnosis | Above Knee |
| S88.019S | ICD 10 Diagnosis | Above Knee |
| S88.029 | ICD 10 Diagnosis | Above Knee |
| S88.029A | ICD 10 Diagnosis | Above Knee |
| S88.029D | ICD 10 Diagnosis | Above Knee |
| S88.029S | ICD 10 Diagnosis | Above Knee |
| S78.019 | ICD 10 Diagnosis | Above Knee |
| S78.019A | ICD 10 Diagnosis | Above Knee |
| S78.019D | ICD 10 Diagnosis | Above Knee |
| S78.019S | ICD 10 Diagnosis | Above Knee |
| S78.029 | ICD 10 Diagnosis | Above Knee |
| S78.029A | ICD 10 Diagnosis | Above Knee |
| S78.029D | ICD 10 Diagnosis | Above Knee |
| S78.029S | ICD 10 Diagnosis | Above Knee |
| S78.119 | ICD 10 Diagnosis | Above Knee |
| S78.119A | ICD 10 Diagnosis | Above Knee |
| S78.119D | ICD 10 Diagnosis | Above Knee |
| S78.119S | ICD 10 Diagnosis | Above Knee |
| S78.129 | ICD 10 Diagnosis | Above Knee |
| S78.129A | ICD 10 Diagnosis | Above Knee |
| S78.129D | ICD 10 Diagnosis | Above Knee |
| S78.129S | ICD 10 Diagnosis | Above Knee |
| S78.919 | ICD 10 Diagnosis | Above Knee |
| S78.919A | ICD 10 Diagnosis | Above Knee |
| S78.919D | ICD 10 Diagnosis | Above Knee |
| S78.919S | ICD 10 Diagnosis | Above Knee |
| S78.929 | ICD 10 Diagnosis | Above Knee |
| S78.929A | ICD 10 Diagnosis | Above Knee |
| S78.929D | ICD 10 Diagnosis | Above Knee |
| S78.929S | ICD 10 Diagnosis | Above Knee |
| Z89.529 | ICD 10 Diagnosis | Above Knee |
| Z89.619 | ICD 10 Diagnosis | Above Knee |
| Z89.629 | ICD 10 Diagnosis | Above Knee |
| 84.16 | ICD 9 Procedure | Above Knee |
| 84.17 | ICD 9 Procedure | Above Knee |
| 84.18 | ICD 9 Procedure | Above Knee |
| 897.2 | ICD 9 Diagnosis | Above Knee |
| 897.3 | ICD 9 Diagnosis | Above Knee |
| 27880 | CPT | Below Knee |
| 27881 | CPT | Below Knee |
| 27882 | CPT | Below Knee |
| 27884 | CPT | Below Knee |
| 27886 | CPT | Below Knee |
| 27888 | CPT | Below Knee |
| 27889 | CPT | Below Knee |
| 0Y6N0Z0 | ICD 10 Procedure | Below Knee |
| 0Y6J0Z1 | ICD 10 Procedure | Below Knee |
| 0Y6J0Z2 | ICD 10 Procedure | Below Knee |
| 0Y6J0Z3 | ICD 10 Procedure | Below Knee |
| 0Y6M0Z0 | ICD 10 Procedure | Below Knee |
| 0Y6H0Z1 | ICD 10 Procedure | Below Knee |
| 0Y6H0Z2 | ICD 10 Procedure | Below Knee |
| 0Y6H0Z3 | ICD 10 Procedure | Below Knee |
| S98.012 | ICD 10 Diagnosis | Below Knee |
| S98.012A | ICD 10 Diagnosis | Below Knee |
| S98.012D | ICD 10 Diagnosis | Below Knee |
| S98.012S | ICD 10 Diagnosis | Below Knee |
| S98.022 | ICD 10 Diagnosis | Below Knee |
| S98.022A | ICD 10 Diagnosis | Below Knee |
| S98.022D | ICD 10 Diagnosis | Below Knee |
| S98.022S | ICD 10 Diagnosis | Below Knee |
| S88.112 | ICD 10 Diagnosis | Below Knee |
| S88.112A | ICD 10 Diagnosis | Below Knee |
| S88.112D | ICD 10 Diagnosis | Below Knee |
| S88.112S | ICD 10 Diagnosis | Below Knee |
| S88.122 | ICD 10 Diagnosis | Below Knee |
| S88.122A | ICD 10 Diagnosis | Below Knee |
| S88.122D | ICD 10 Diagnosis | Below Knee |
| S88.122S | ICD 10 Diagnosis | Below Knee |
| S88.912 | ICD 10 Diagnosis | Below Knee |
| S88.912A | ICD 10 Diagnosis | Below Knee |
| S88.912D | ICD 10 Diagnosis | Below Knee |
| S88.912S | ICD 10 Diagnosis | Below Knee |
| S88.922 | ICD 10 Diagnosis | Below Knee |
| S88.922A | ICD 10 Diagnosis | Below Knee |
| S88.922D | ICD 10 Diagnosis | Below Knee |
| S88.922S | ICD 10 Diagnosis | Below Knee |
| Z89.432 | ICD 10 Diagnosis | Below Knee |
| Z89.442 | ICD 10 Diagnosis | Below Knee |
| Z89.512 | ICD 10 Diagnosis | Below Knee |
| S98.011 | ICD 10 Diagnosis | Below Knee |
| S98.011A | ICD 10 Diagnosis | Below Knee |
| S98.011D | ICD 10 Diagnosis | Below Knee |
| S98.011S | ICD 10 Diagnosis | Below Knee |
| S98.021 | ICD 10 Diagnosis | Below Knee |
| S98.021A | ICD 10 Diagnosis | Below Knee |
| S98.021D | ICD 10 Diagnosis | Below Knee |
| S98.021S | ICD 10 Diagnosis | Below Knee |
| S88.111 | ICD 10 Diagnosis | Below Knee |
| S88.111A | ICD 10 Diagnosis | Below Knee |
| S88.111D | ICD 10 Diagnosis | Below Knee |
| S88.111S | ICD 10 Diagnosis | Below Knee |
| S88.121 | ICD 10 Diagnosis | Below Knee |
| S88.121A | ICD 10 Diagnosis | Below Knee |
| S88.121D | ICD 10 Diagnosis | Below Knee |
| S88.121S | ICD 10 Diagnosis | Below Knee |
| S88.911 | ICD 10 Diagnosis | Below Knee |
| S88.911A | ICD 10 Diagnosis | Below Knee |
| S88.911D | ICD 10 Diagnosis | Below Knee |
| S88.911S | ICD 10 Diagnosis | Below Knee |
| S88.921 | ICD 10 Diagnosis | Below Knee |
| S88.921A | ICD 10 Diagnosis | Below Knee |
| S88.921D | ICD 10 Diagnosis | Below Knee |
| S88.921S | ICD 10 Diagnosis | Below Knee |
| Z89.431 | ICD 10 Diagnosis | Below Knee |
| Z89.441 | ICD 10 Diagnosis | Below Knee |
| Z89.511 | ICD 10 Diagnosis | Below Knee |
| S98.019 | ICD 10 Diagnosis | Below Knee |
| S98.019A | ICD 10 Diagnosis | Below Knee |
| S98.019D | ICD 10 Diagnosis | Below Knee |
| S98.019S | ICD 10 Diagnosis | Below Knee |
| S98.029 | ICD 10 Diagnosis | Below Knee |
| S98.029A | ICD 10 Diagnosis | Below Knee |
| S98.029D | ICD 10 Diagnosis | Below Knee |
| S98.029S | ICD 10 Diagnosis | Below Knee |
| S88.119 | ICD 10 Diagnosis | Below Knee |
| S88.119A | ICD 10 Diagnosis | Below Knee |
| S88.119D | ICD 10 Diagnosis | Below Knee |
| S88.119S | ICD 10 Diagnosis | Below Knee |
| S88.129 | ICD 10 Diagnosis | Below Knee |
| S88.129A | ICD 10 Diagnosis | Below Knee |
| S88.129D | ICD 10 Diagnosis | Below Knee |
| S88.129S | ICD 10 Diagnosis | Below Knee |
| S88.919 | ICD 10 Diagnosis | Below Knee |
| S88.919A | ICD 10 Diagnosis | Below Knee |
| S88.919D | ICD 10 Diagnosis | Below Knee |
| S88.919S | ICD 10 Diagnosis | Below Knee |
| S88.929 | ICD 10 Diagnosis | Below Knee |
| S88.929A | ICD 10 Diagnosis | Below Knee |
| S88.929D | ICD 10 Diagnosis | Below Knee |
| S88.929S | ICD 10 Diagnosis | Below Knee |
| Z89.439 | ICD 10 Diagnosis | Below Knee |
| Z89.449 | ICD 10 Diagnosis | Below Knee |
| Z89.519 | ICD 10 Diagnosis | Below Knee |
| 84.13 | ICD 9 Procedure | Below Knee |
| 84.14 | ICD 9 Procedure | Below Knee |
| 84.15 | ICD 9 Procedure | Below Knee |
| 897.0 | ICD 9 Diagnosis | Below Knee |
| 897.1 | ICD 9 Diagnosis | Below Knee |
| 28800 | CPT | Partial Foot |
| 28805 | CPT | Partial Foot |
| 28810 | CPT | Partial Foot |
| 28820 | CPT | Partial Foot |
| 28825 | CPT | Partial Foot |
| 0Y6Y0Z0 | ICD 10 Procedure | Partial Foot |
| 0Y6Y0Z1 | ICD 10 Procedure | Partial Foot |
| 0Y6Y0Z2 | ICD 10 Procedure | Partial Foot |
| 0Y6Y0Z3 | ICD 10 Procedure | Partial Foot |
| 0Y6W0Z0 | ICD 10 Procedure | Partial Foot |
| 0Y6W0Z1 | ICD 10 Procedure | Partial Foot |
| 0Y6W0Z2 | ICD 10 Procedure | Partial Foot |
| 0Y6W0Z3 | ICD 10 Procedure | Partial Foot |
| 0Y6U0Z0 | ICD 10 Procedure | Partial Foot |
| 0Y6U0Z1 | ICD 10 Procedure | Partial Foot |
| 0Y6U0Z2 | ICD 10 Procedure | Partial Foot |
| 0Y6U0Z3 | ICD 10 Procedure | Partial Foot |
| 0Y6S0Z0 | ICD 10 Procedure | Partial Foot |
| 0Y6S0Z1 | ICD 10 Procedure | Partial Foot |
| 0Y6S0Z2 | ICD 10 Procedure | Partial Foot |
| 0Y6S0Z3 | ICD 10 Procedure | Partial Foot |
| 0Y6Q0Z0 | ICD 10 Procedure | Partial Foot |
| 0Y6Q0Z1 | ICD 10 Procedure | Partial Foot |
| 0Y6Q0Z2 | ICD 10 Procedure | Partial Foot |
| 0Y6Q0Z3 | ICD 10 Procedure | Partial Foot |
| 0Y6N0Z4 | ICD 10 Procedure | Partial Foot |
| 0Y6N0Z5 | ICD 10 Procedure | Partial Foot |
| 0Y6N0Z6 | ICD 10 Procedure | Partial Foot |
| 0Y6N0Z7 | ICD 10 Procedure | Partial Foot |
| 0Y6N0Z8 | ICD 10 Procedure | Partial Foot |
| 0Y6N0Z9 | ICD 10 Procedure | Partial Foot |
| 0Y6N0ZB | ICD 10 Procedure | Partial Foot |
| 0Y6N0ZC | ICD 10 Procedure | Partial Foot |
| 0Y6N0ZD | ICD 10 Procedure | Partial Foot |
| 0Y6N0ZF | ICD 10 Procedure | Partial Foot |
| 0Y6V0Z0 | ICD 10 Procedure | Partial Foot |
| 0Y6V0Z1 | ICD 10 Procedure | Partial Foot |
| 0Y6V0Z2 | ICD 10 Procedure | Partial Foot |
| 0Y6V0Z3 | ICD 10 Procedure | Partial Foot |
| 0Y6X0Z0 | ICD 10 Procedure | Partial Foot |
| 0Y6X0Z1 | ICD 10 Procedure | Partial Foot |
| 0Y6X0Z2 | ICD 10 Procedure | Partial Foot |
| 0Y6X0Z3 | ICD 10 Procedure | Partial Foot |
| 0Y6V0Z0 | ICD 10 Procedure | Partial Foot |
| 0Y6V0Z1 | ICD 10 Procedure | Partial Foot |
| 0Y6V0Z2 | ICD 10 Procedure | Partial Foot |
| 0Y6V0Z3 | ICD 10 Procedure | Partial Foot |
| 0Y6V0Z0 | ICD 10 Procedure | Partial Foot |
| 0Y6V0Z1 | ICD 10 Procedure | Partial Foot |
| 0Y6V0Z2 | ICD 10 Procedure | Partial Foot |
| 0Y6V0Z3 | ICD 10 Procedure | Partial Foot |
| 0Y6T0Z0 | ICD 10 Procedure | Partial Foot |
| 0Y6T0Z1 | ICD 10 Procedure | Partial Foot |
| 0Y6T0Z2 | ICD 10 Procedure | Partial Foot |
| 0Y6T0Z3 | ICD 10 Procedure | Partial Foot |
| 0Y6R0Z0 | ICD 10 Procedure | Partial Foot |
| 0Y6R0Z1 | ICD 10 Procedure | Partial Foot |
| 0Y6R0Z2 | ICD 10 Procedure | Partial Foot |
| 0Y6R0Z3 | ICD 10 Procedure | Partial Foot |
| 0Y6P0Z0 | ICD 10 Procedure | Partial Foot |
| 0Y6P0Z1 | ICD 10 Procedure | Partial Foot |
| 0Y6P0Z2 | ICD 10 Procedure | Partial Foot |
| 0Y6P0Z3 | ICD 10 Procedure | Partial Foot |
| 0Y6M0Z4 | ICD 10 Procedure | Partial Foot |
| 0Y6M0Z5 | ICD 10 Procedure | Partial Foot |
| 0Y6M0Z6 | ICD 10 Procedure | Partial Foot |
| 0Y6M0Z7 | ICD 10 Procedure | Partial Foot |
| 0Y6M0Z8 | ICD 10 Procedure | Partial Foot |
| 0Y6M0Z9 | ICD 10 Procedure | Partial Foot |
| 0Y6M0ZB | ICD 10 Procedure | Partial Foot |
| 0Y6M0ZC | ICD 10 Procedure | Partial Foot |
| 0Y6M0ZD | ICD 10 Procedure | Partial Foot |
| 0Y6M0ZF | ICD 10 Procedure | Partial Foot |
| S98.112 | ICD 10 Diagnosis | Partial Foot |
| S98.112A | ICD 10 Diagnosis | Partial Foot |
| S98.112D | ICD 10 Diagnosis | Partial Foot |
| S98.112S | ICD 10 Diagnosis | Partial Foot |
| S98.122 | ICD 10 Diagnosis | Partial Foot |
| S98.122A | ICD 10 Diagnosis | Partial Foot |
| S98.122D | ICD 10 Diagnosis | Partial Foot |
| S98.122S | ICD 10 Diagnosis | Partial Foot |
| S98.132 | ICD 10 Diagnosis | Partial Foot |
| S98.132A | ICD 10 Diagnosis | Partial Foot |
| S98.132D | ICD 10 Diagnosis | Partial Foot |
| S98.132S | ICD 10 Diagnosis | Partial Foot |
| S98.142 | ICD 10 Diagnosis | Partial Foot |
| S98.142A | ICD 10 Diagnosis | Partial Foot |
| S98.142D | ICD 10 Diagnosis | Partial Foot |
| S98.142S | ICD 10 Diagnosis | Partial Foot |
| S98.212 | ICD 10 Diagnosis | Partial Foot |
| S98.212A | ICD 10 Diagnosis | Partial Foot |
| S98.212D | ICD 10 Diagnosis | Partial Foot |
| S98.212S | ICD 10 Diagnosis | Partial Foot |
| S98.222 | ICD 10 Diagnosis | Partial Foot |
| S98.222A | ICD 10 Diagnosis | Partial Foot |
| S98.222D | ICD 10 Diagnosis | Partial Foot |
| S98.222S | ICD 10 Diagnosis | Partial Foot |
| S98.312 | ICD 10 Diagnosis | Partial Foot |
| S98.312A | ICD 10 Diagnosis | Partial Foot |
| S98.312D | ICD 10 Diagnosis | Partial Foot |
| S98.312S | ICD 10 Diagnosis | Partial Foot |
| S98.322 | ICD 10 Diagnosis | Partial Foot |
| S98.322A | ICD 10 Diagnosis | Partial Foot |
| S98.322D | ICD 10 Diagnosis | Partial Foot |
| S98.322S | ICD 10 Diagnosis | Partial Foot |
| S98.912 | ICD 10 Diagnosis | Partial Foot |
| S98.912A | ICD 10 Diagnosis | Partial Foot |
| S98.912D | ICD 10 Diagnosis | Partial Foot |
| S98.912S | ICD 10 Diagnosis | Partial Foot |
| S98.922 | ICD 10 Diagnosis | Partial Foot |
| S98.922A | ICD 10 Diagnosis | Partial Foot |
| S98.922D | ICD 10 Diagnosis | Partial Foot |
| S98.922S | ICD 10 Diagnosis | Partial Foot |
| Z89.412 | ICD 10 Diagnosis | Partial Foot |
| Z89.422 | ICD 10 Diagnosis | Partial Foot |
| S98.111 | ICD 10 Diagnosis | Partial Foot |
| S98.111A | ICD 10 Diagnosis | Partial Foot |
| S98.111D | ICD 10 Diagnosis | Partial Foot |
| S98.111S | ICD 10 Diagnosis | Partial Foot |
| S98.121 | ICD 10 Diagnosis | Partial Foot |
| S98.121A | ICD 10 Diagnosis | Partial Foot |
| S98.121D | ICD 10 Diagnosis | Partial Foot |
| S98.121S | ICD 10 Diagnosis | Partial Foot |
| S98.131 | ICD 10 Diagnosis | Partial Foot |
| S98.131A | ICD 10 Diagnosis | Partial Foot |
| S98.131D | ICD 10 Diagnosis | Partial Foot |
| S98.131S | ICD 10 Diagnosis | Partial Foot |
| S98.141 | ICD 10 Diagnosis | Partial Foot |
| S98.141A | ICD 10 Diagnosis | Partial Foot |
| S98.141D | ICD 10 Diagnosis | Partial Foot |
| S98.141S | ICD 10 Diagnosis | Partial Foot |
| S98.211 | ICD 10 Diagnosis | Partial Foot |
| S98.211A | ICD 10 Diagnosis | Partial Foot |
| S98.211D | ICD 10 Diagnosis | Partial Foot |
| S98.211S | ICD 10 Diagnosis | Partial Foot |
| S98.221 | ICD 10 Diagnosis | Partial Foot |
| S98.221A | ICD 10 Diagnosis | Partial Foot |
| S98.221D | ICD 10 Diagnosis | Partial Foot |
| S98.221S | ICD 10 Diagnosis | Partial Foot |
| S98.311 | ICD 10 Diagnosis | Partial Foot |
| S98.311A | ICD 10 Diagnosis | Partial Foot |
| S98.311D | ICD 10 Diagnosis | Partial Foot |
| S98.311S | ICD 10 Diagnosis | Partial Foot |
| S98.321 | ICD 10 Diagnosis | Partial Foot |
| S98.321A | ICD 10 Diagnosis | Partial Foot |
| S98.321D | ICD 10 Diagnosis | Partial Foot |
| S98.321S | ICD 10 Diagnosis | Partial Foot |
| S98.911 | ICD 10 Diagnosis | Partial Foot |
| S98.911A | ICD 10 Diagnosis | Partial Foot |
| S98.911D | ICD 10 Diagnosis | Partial Foot |
| S98.911S | ICD 10 Diagnosis | Partial Foot |
| S98.921 | ICD 10 Diagnosis | Partial Foot |
| S98.921A | ICD 10 Diagnosis | Partial Foot |
| S98.921D | ICD 10 Diagnosis | Partial Foot |
| S98.921S | ICD 10 Diagnosis | Partial Foot |
| Z89.411 | ICD 10 Diagnosis | Partial Foot |
| Z89.421 | ICD 10 Diagnosis | Partial Foot |
| S98.119 | ICD 10 Diagnosis | Partial Foot |
| S98.119A | ICD 10 Diagnosis | Partial Foot |
| S98.119D | ICD 10 Diagnosis | Partial Foot |
| S98.119S | ICD 10 Diagnosis | Partial Foot |
| S98.129 | ICD 10 Diagnosis | Partial Foot |
| S98.129A | ICD 10 Diagnosis | Partial Foot |
| S98.129D | ICD 10 Diagnosis | Partial Foot |
| S98.129S | ICD 10 Diagnosis | Partial Foot |
| S98.139 | ICD 10 Diagnosis | Partial Foot |
| S98.139A | ICD 10 Diagnosis | Partial Foot |
| S98.139D | ICD 10 Diagnosis | Partial Foot |
| S98.139S | ICD 10 Diagnosis | Partial Foot |
| S98.149 | ICD 10 Diagnosis | Partial Foot |
| S98.149A | ICD 10 Diagnosis | Partial Foot |
| S98.149D | ICD 10 Diagnosis | Partial Foot |
| S98.149S | ICD 10 Diagnosis | Partial Foot |
| S98.219 | ICD 10 Diagnosis | Partial Foot |
| S98.219A | ICD 10 Diagnosis | Partial Foot |
| S98.219D | ICD 10 Diagnosis | Partial Foot |
| S98.219S | ICD 10 Diagnosis | Partial Foot |
| S98.229 | ICD 10 Diagnosis | Partial Foot |
| S98.229A | ICD 10 Diagnosis | Partial Foot |
| S98.229D | ICD 10 Diagnosis | Partial Foot |
| S98.229S | ICD 10 Diagnosis | Partial Foot |
| S98.319 | ICD 10 Diagnosis | Partial Foot |
| S98.319A | ICD 10 Diagnosis | Partial Foot |
| S98.319D | ICD 10 Diagnosis | Partial Foot |
| S98.319S | ICD 10 Diagnosis | Partial Foot |
| S98.329 | ICD 10 Diagnosis | Partial Foot |
| S98.329A | ICD 10 Diagnosis | Partial Foot |
| S98.329D | ICD 10 Diagnosis | Partial Foot |
| S98.329S | ICD 10 Diagnosis | Partial Foot |
| S98.919 | ICD 10 Diagnosis | Partial Foot |
| S98.919A | ICD 10 Diagnosis | Partial Foot |
| S98.919D | ICD 10 Diagnosis | Partial Foot |
| S98.919S | ICD 10 Diagnosis | Partial Foot |
| S98.929 | ICD 10 Diagnosis | Partial Foot |
| S98.929A | ICD 10 Diagnosis | Partial Foot |
| S98.929D | ICD 10 Diagnosis | Partial Foot |
| S98.929S | ICD 10 Diagnosis | Partial Foot |
| Z89.419 | ICD 10 Diagnosis | Partial Foot |
| Z89.429 | ICD 10 Diagnosis | Partial Foot |
| 84.11 | ICD 9 Procedure | Partial Foot |
| 84.12 | ICD 9 Procedure | Partial Foot |
| 895.0 | ICD 9 Diagnosis | Partial Foot |
| 895.1 | ICD 9 Diagnosis | Partial Foot |
| 896.0 | ICD 9 Diagnosis | Partial Foot |
| 896.1 | ICD 9 Diagnosis | Partial Foot |
