# Supplementary material for: Trends in incidence and correlation between medical costs and lost workdays for work‐related amputations in the State of California from 2007 to 2018
Source: Health Sci Rep. 2021 Jul 1;4(3):e319. doi: 10.1002/hsr2.319 (PMC8247939; doi:10.1002/hsr2.319)
Supplement: Supplementary file 2 — Table S2. Annual proportion and average incidence of amputations according to industry and nature of injury from 2007 to 2018. [file HSR2-4-e319-s002.docx]

Supplementary 2 Annual proportion and average incidence of amputations according to industry and nature of injury from 2007-2018

| Row ID |  | 2007 | 2008 | 2009 | 2010 | 2011 | 2012 |
| --- | --- | --- | --- | --- | --- | --- | --- |
| 1 | Industry |  |  |  |  |  |  |
| 2 | Construction | 141(9.9%) | 361(14.7%) | 189(11.7%) | 138(10.7%) | 137(11.6%) | 172(13.9%) |
| 3 | Manufacturing | 368(25.8%) | 501(20.3%) | 315(19.5%) | 294(22.8%) | 327(27.7%) | 321(25.9%) |
| 4 | Retail Trade | 103(7.2%) | 199(8.1%) | 145(9.0%) | 118(9.1%) | 119(10.1%) | 137(11.1%) |
| 5 | Administrative and Support and Waste Management and Remediation Services | 83(5.8%) | 150(6.1%) | 117(7.3%) | 102(7.9%) | 73(6.2%) | 97(7.8%) |
| 6 | Agriculture, Forestry, Fishing and Hunting | 41(2.9%) | 99(4.0%) | 66(4.1%) | 46(3.6%) | 41(3.5%) | 51(4.1%) |
| 7 | Wholesale Trade | 69(4.8%) | 63(2.6%) | 58(3.6%) | 62(4.8%) | 56(4.7%) | 54(4.4%) |
| 8 | Accommodation and Food Services | 34(2.4%) | 56(2.3%) | 35(2.2%) | 46(3.6%) | 47(4.0%) | 53(4.3%) |
| 9 | Transportation and Warehousing | 49(3.4%) | 95(3.9%) | 54(3.3%) | 40(3.1%) | 49(4.2%) | 49(4.0%) |
| 10 | Arts, Entertainment, and Recreation | 37(2.6%) | 64(2.6%) | 42(2.6%) | 73(5.7%) | 32(2.7%) | 45(3.6%) |
| 11 | Other Services (except Public Administration) | 25(1.8%) | 44(1.8%) | 38(2.4%) | 39(3.0%) | 31(2.6%) | 36(2.9%) |
| 12 | Professional, Scientific, and Technical Services | 49(3.4%) | 40(1.6%) | 48(3.0%) | 42(3.3%) | 37(3.1%) | 38(3.1%) |
| 13 | Public Administration | 22(1.5%) | 68(2.8%) | 36(2.2%) | 21(1.6%) | 34(2.9%) | 28(2.3%) |
| 14 | Other (<18%)^c^ | 404(28.4%) | 724(29.4%) | 469(29.1%) | 271(21.0%) | 196(16.6%) | 158(12.8%) |
| 15 | Nature of Injury |  |  |  |  |  |  |
| 16 | Laceration | 395(27.7%) | 744(30.2%) | 456(28.3%) | 378(29.3%) | 348(29.5%) | 385(31.1%) |
| 17 | Amputation | 361(25.3%) | 675(27.4%) | 400(24.8%) | 323(25.0%) | 327(27.7%) | 319(25.7%) |
| 18 | Fracture | 93(6.5%) | 198(8.0%) | 108(6.7%) | 75(5.8%) | 87(7.4%) | 112(9.0%) |
| 19 | Crushing | 117(8.2%) | 185(7.5%) | 118(7.3%) | 86(6.7%) | 89(7.5%) | 83(6.7%) |
| 20 | Strain or Tear | 95(6.7%) | 143(5.8%) | 142(8.8%) | 128(9.9%) | 93(7.9%) | 82(6.6%) |
| 21 | All Other Specific Injuries, NOC | 69(4.8%) | 98(4.0%) | 66(4.1%) | 54(4.2%) | 44(3.7%) | 42(3.4%) |
| 22 | Severance | 79(5.5%) | 104(4.2%) | 67(4.2%) | 45(3.5%) | 49(4.2%) | 44(3.6%) |
| 23 | Contusion | 63(4.4%) | 70(2.8%) | 56(3.5%) | 44(3.4%) | 34(2.9%) | 51(4.1%) |
| 24 | Sprain or Tear | 34(2.4%) | 60(2.4%) | 52(3.2%) | 43(3.3%) | 21(1.8%) | 26(2.1%) |
| 25 | Multiple Physical Injuries Only | 28(2.0) | 30(1.2%) | 25(1.6%) | 31(2.4%) | 15(1.3%) | 19(1.5%) |
| 26 | All Other Cumulative Injuries | 16(1.1%) | 44(1.8%) | 30(1.9%) | 21(1.6%) | 21(1.8%) | 24(1.9%) |
| 27 | Other (<5%)^c^ | 75(5.3%) | 113(4.6%) | 92(5.7%) | 64(5.0%) | 51(4.3%) | 52(4.2%) |
| ^a^ Industry incidence per 100,000 workers and calculated with respect to 12-year average worker population cited for those specific industries.  ^b^ Nature of injury incidence per 1,000,000 workers and calculated with respect to total 12-year average worker population.  ^c^ contains claims with missing data | | | | | | | |

| Row ID | 2013 | 2014 | 2015 | 2016 | 2017 | 2018 | Average | Incidence^a,b^ |
| --- | --- | --- | --- | --- | --- | --- | --- | --- |
| 1 |  |  |  |  |  |  |  |  |
| 2 | 129(11.8%) | 188(14.2%) | 199(14.7%) | 151(13.2%) | 209(15.2%) | 226(15.8%) | 186.7(13.2%) | 26.0(22.4-30.0) |
| 3 | 276(25.2%) | 325(24.5%) | 354(26.1%) | 293(25.7%) | 346(25.2%) | 357(25.0%) | 339.8(24.1%) | 25.9(23.3-28.9) |
| 4 | 112(10.2%) | 147(11.1%) | 127(9.4%) | 129(11.3%) | 125(9.1%) | 126(8.8%) | 132.3(9.4%) | 8.3(6.9-9.8) |
| 5 | 119(10.9%) | 147(11.1%) | 148(10.9%) | 115(10.1%) | 133(9.7%) | 154(10.8%) | 119.8(8.5%) | 12.0(10.0-14.4) |
| 6 | 53(4.8%) | 87(6.6%) | 88(6.5%) | 91(8.0%) | 76(5.5%) | 92(6.4%) | 69.3(4.9%) | 15.1(11.8-19.2) |
| 7 | 56(5.1%) | 53(4.0%) | 61(4.5%) | 53(4.6%) | 69(5.0%) | 67(4.7%) | 60.1(4.3%) | 8.7(6.7-11.2) |
| 8 | 69(6.3%) | 82(6.2%) | 82(6.0%) | 53(4.6%) | 68(5.0%) | 94(6.6%) | 59.9(4.2%) | 4.2(3.2-5.4) |
| 9 | 51(4.7%) | 59(4.4%) | 50(3.7%) | 40(3.5%) | 65(4.7%) | 52(3.6%) | 54.4(3.9%) | 11.8(9.0-15.5) |
| 10 | 47(4.3%) | 32(2.4%) | 28(2.1%) | 33(2.9%) | 32(2.3%) | 30(2.1%) | 41.3(2.9%) | 15.1(11.0-20.7) |
| 11 | 38(3.5%) | 33(2.5%) | 43(3.2%) | 32(2.8%) | 47(3.4%) | 48(3.4%) | 37.8(2.7%) | 6.0(4.3-8.3) |
| 12 | 34(3.1%) | 40(3.0%) | 30(2.2%) | 25(2.2%) | 28(2.0%) | 27(1.9%) | 36.5(2.6%) | 3.2(2.3-4.5) |
| 13 | 24(2.2%) | 27(2.0%) | 34(2.5%) | 38(3.3%) | 40(2.9%) | 30(2.1%) | 33.5(2.4%) | 1.4(1.0-2.0) |
| 14 | 88(8.0%) | 106(8.0%) | 113(8.3%) | 89(7.8%) | 133(9.7%) | 125(8.8%) | 239.7(17.0%) | --- |
| 15 |  |  |  |  |  |  |  |  |
| 16 | 359(32.8%) | 436(32.9%) | 415(30.6%) | 359(31.4%) | 383(27.9%) | 410(28.7%) | 422.3(29.9%) | 26.8(24.3-29.5) |
| 17 | 307(28.0%) | 361(27.2%) | 378(27.9%) | 296(25.9%) | 351(25.6%) | 427(29.9%) | 377.1(26.7%) | 23.9(21.6-26.5) |
| 18 | 82(7.5%) | 104(7.8%) | 107(7.9%) | 84(7.4%) | 104(7.6%) | 117(8.2%) | 105.9(7.5%) | 6.7(5.5-8.2) |
| 19 | 64(5.8%) | 105(7.9%) | 112(8.3%) | 95(8.3%) | 103(7.5%) | 113(7.9%) | 105.8(7.5%) | 6.7(5.5-8.1) |
| 20 | 50(4.6%) | 74(5.6%) | 58(4.3%) | 77(6.7%) | 98(7.1%) | 77(5.4%) | 93.1(6.6%) | 5.9(4.8-7.3) |
| 21 | 43(3.9%) | 52(3.9%) | 49(3.6%) | 50(4.4%) | 81(5.9%) | 85(6.0%) | 61.1(4.3%) | 3.9(3.0-5.0) |
| 22 | 34(3.1%) | 37(2.8%) | 60(4.4%) | 37(3.2%) | 61(4.4%) | 45(3.2%) | 55.2(3.9%) | 3.5(2.7-4.6) |
| 23 | 47(4.3%) | 48(3.6%) | 49(3.6%) | 40(3.5%) | 48(3.5%) | 34(2.4%) | 48.7(3.5%) | 3.1(2.3-4.1) |
| 24 | 23(2.1%) | 17(1.3%) | 26(1.9%) | 14(1.2%) | 23(1.7%) | 15(1.1%) | 29.5(2.1%) | 1.9(1.3-2.7) |
| 25 | 21(1.9%) | 26(2.0%) | 28(2.1%) | 24(2.1%) | 30(2.2%) | 27(1.9%) | 25.3(1.8%) | 1.6(1.1-2.4) |
| 26 | 15(1.4%) | 18(1.4%) | 30(2.2%) | 14(1.2%) | 14(1.0%) | 16(1.1%) | 21.9(1.6%) | 1.4(0.9-2.1) |
| 27 | 51(4.7%) | 48(3.6%) | 45(3.3%) | 52(4.6%) | 75(5.5%) | 62(4.3%) | 65(4.6%) | --- |
