# Supplementary material for: Trends in incidence and correlation between medical costs and lost workdays for work‐related amputations in the State of California from 2007 to 2018
Source: Health Sci Rep. 2021 Jul 1;4(3):e319. doi: 10.1002/hsr2.319 (PMC8247939; doi:10.1002/hsr2.319)
Supplement: Supplementary file 3 — Table S3. Complete annual demographics of persons who experienced a work‐related amputation in California from 2007 to 2018. [file HSR2-4-e319-s001.docx]

Supplementary 3 Complete annual demographics of persons who experienced a work-related amputation in California from 2007-2018

| Row ID |  | 2007 | 2008 | 2009 | 2010 | 2011 | 2012 |
| --- | --- | --- | --- | --- | --- | --- | --- |
| 1 | # of Claims | 1425 | 2464 | 1612 | 1292 | 1179 | 1239 |
| 2 | Working Population | 15747249 | 15527344 | 14498327 | 14475411 | 14697321 | 15113718 |
| 3 | Incidence (95% CI)^a^ | 9.1(8.6-9.5) | 15.9(15.3-16.5) | 11.1(10.6-11.7) | 8.9(8.5-9.4) | 8.0(7.6-8.5) | 8.2(7.8-8.7) |
| 4 | Age^b^ |  |  |  |  |  |  |
| 5 | 16-24 | 207(14.5%) | 335(13.6%) | 169(10.5%) | 123(9.5%) | 103(8.7%) | 126(10.2%) |
| 6 | 25-34 | 309(21.7%) | 586(23.8%) | 389(24.1%) | 288(22.3%) | 274(23.2%) | 266(21.5%) |
| 7 | 35-44 | 339(23.8%) | 638(25.9%) | 368(22.8%) | 303(23.5%) | 294(24.9%) | 279(22.5%) |
| 8 | 45-54 | 356(25.0%) | 564(22.9%) | 398(24.7%) | 343(26.5%) | 297(25.2%) | 324(26.2%) |
| 9 | 55-64 | 181(12.7%) | 277(11.2%) | 250(15.5%) | 206(15.9%) | 184(15.6%) | 217(17.5%) |
| 10 | 65+ | 33(2.3%) | 64(2.6%) | 38(2.4%) | 29(2.2%) | 27(2.3%) | 27(2.2%) |
| 11 | Gender |  |  |  |  |  |  |
| 12 | Male | 1184(83.1%) | 2134(86.6%) | 1341(83.2%) | 1068(82.7%) | 1001(84.9%) | 1051(84.8%) |
| 13 | Female | 234(16.4%) | 321(13.0%) | 267(16.6%) | 222(17.2%) | 176(14.9%) | 187(15.1%) |
| 14 | Annual Salary ($)^b^ |  |  |  |  |  |  |
| 15 | 0-24,999 | 589(41.3%) | 950(38.6%) | 592(36.7%) | 494(38.2%) | 455(38.6%) | 458(37.0%) |
| 16 | 25,000-34,999 | 339(23.8%) | 568(23.1%) | 372(23.1%) | 279(21.6%) | 247(20.9%) | 276(22.3%) |
| 17 | 35,000-44,999 | 177(12.4%) | 351(14.2%) | 220(13.6%) | 182(14.1%) | 168(14.2%) | 161(13.0%) |
| 18 | 45,000-54,999 | 131(9.2%) | 231(9.4%) | 167(10.4%) | 126(9.8%) | 120(10.2%) | 129(10.4%) |
| 19 | 55,000-64,999 | 60(4.2%) | 123(5.0%) | 86(5.3%) | 68(5.3%) | 67(5.7%) | 68(5.5%) |
| 20 | 65,000-74,000 | 74(5.2%) | 112(4.5%) | 83(5.1%) | 54(4.2%) | 49(4.2%) | 44(3.6%) |
| 21 | 75,000+ | 54(3.8%) | 125(5.1%) | 89(5.5%) | 85(6.6%) | 71(6.0%) | 100(8.1%) |
| 22 | Prosthesis Use | 50(3.5%) | 90(3.7%) | 43(2.7%) | 51(3.9%) | 53(4.5%) | 44(3.6%) |
| 23 | Presence of Lawsuit | 358(25.1%) | 355(14.4%) | 314(19.5%) | 245(19.0%) | 240(20.4%) | 262(21.1%) |
| 24 | Employment Status |  |  |  |  |  |  |
| 25 | Full-Time | 1142(80.1%) | 1953(79.3%) | 1189(73.8%) | 958(74.1%) | 902(76.5%) | 825(66.6%) |
| 26 | Part-Time | 95(6.7%) | 146(5.9%) | 119(7.4%) | 98(7.6%) | 102(8.7%) | 93(7.5%) |
| 27 | Other | 188(13.2%) | 365(14.8%) | 304(18.9%) | 236(18.3%) | 175(14.8%) | 321(25.9%) |
| 28 | Medical Complexity |  |  |  |  |  |  |
| 29 | Medical Visits^b^ | 5\|**8**\|13 | 5\|**8**\|13 | 5\|**8**\|13 | 5\|**9**\|14 | 4\|**8**\|13 | 4\|**8**\|12 |
| 30 | Unique Diagnoses^b^ | 13\|**21**\|30 | 13\|**21**\|31 | 12\|**21**\|31 | 12\|**22**\|34\| | 10\|**21**\|33 | 11\|**20**\|31 |
| ^a^ per 100,000 workers  ^b^ 25^th^\|**50^th^**\|75^th^ percentile values | | | | | | | |

| Row ID | 2013 | 2014 | 2015 | 2016 | 2017 | 2018 | Average |
| --- | --- | --- | --- | --- | --- | --- | --- |
| 1 | 1096 | 1326 | 1357 | 1142 | 1371 | 1428 | 1410.9 |
| 2 | 15543166 | 15992052 | 16481383 | 16872901 | 17064414 | 17386502 | 15783315.7 |
| 3 | 7.1(6.6-7.5) | 8.3(7.9-8.8) | 8.2(7.8-8.7) | 6.8(6.4-7.2) | 8.0(7.6-8.5) | 8.2(7.8-8.7) | 8.9(8.5-9.4) |
| 4 |  |  |  |  |  |  | 30\|**42**\|52 |
| 5 | 154(14.1%) | 166(12.5%) | 144(10.6%) | 109(9.5%) | 135(9.8%) | 155(10.9%) | 160.5(11.4%) |
| 6 | 243(22.2%) | 299(22.5%) | 270(19.9%) | 279(24.4%) | 293(21.4%) | 325(22.8%) | 318.4(22.6%) |
| 7 | 246(22.4%) | 295(22.2%) | 304(22.4%) | 235(20.6%) | 260(19.0%) | 264(18.5%) | 318.8(22.6%) |
| 8 | 262(23.9%) | 302(22.8%) | 319(23.5%) | 263(23.0%) | 332(24.2%) | 353(24.7%) | 342.8(24.3%) |
| 9 | 157(14.3%) | 223(16.8%) | 265(19.5%) | 210(18.4%) | 270(19.7%) | 260(18.2%) | 225.0(15.9%) |
| 10 | 34(3.1%) | 41(3.1%) | 55(4.1%) | 42(3.7%) | 64(4.7%) | 60(4.2%) | 42.8(3.0%) |
| 11 |  |  |  |  |  |  |  |
| 12 | 933(85.1%) | 1159(87.4%) | 1179(86.9%) | 976(85.5%) | 1135(82.8%) | 1204(84.3%) | 1197.1(84.8%) |
| 13 | 157(14.3%) | 164(12.4%) | 174(12.8%) | 162(14.2%) | 226(16.5%) | 213(14.9%) | 208.6(14.8%) |
| 14 |  |  |  |  |  |  | 21630\|**31200**\|47482 |
| 15 | 430(39.2%) | 486(36.7%) | 457(33.7%) | 314(27.5%) | 301(22.0%) | 276(19.3%) | 483.5(34.3%) |
| 16 | 245(22.4%) | 291(21.9%) | 291(21.4%) | 290(25.4%) | 343(25.0%) | 368(25.8%) | 325.8(23.1%) |
| 17 | 151(13.8%) | 197(14.9%) | 219(16.1%) | 191(16.7%) | 240(17.5%) | 276(19.3%) | 211.1(15.0%) |
| 18 | 106(9.7%) | 123(9.3%) | 131(9.7%) | 118(10.3%) | 154(11.2%) | 181(12.7%) | 143.1(10.1%) |
| 19 | 63(5.7%) | 68(5.1%) | 87(6.4%) | 73(6.4%) | 106(7.7%) | 104(7.3%) | 81.1(5.7%) |
| 20 | 39(3.6%) | 54(4.1%) | 55(4.1%) | 48(4.2%) | 52(3.8%) | 59(4.1%) | 60.3(4.3%) |
| 21 | 62(5.7%) | 107(8.1%) | 117(8.6%) | 108(9.5%) | 174(12.7%) | 163(11.4%) | 104.6(7.4%) |
| 22 | 48(4.4%) | 43(3.2%) | 54(4.0%) | 52(4.6%) | 55(4.0%) | 36(2.5%) | 51.6(3.7%) |
| 23 | 261(23.8%) | 247(18.6%) | 251(18.5%) | 162(14.2%) | 292(21.3%) | 262(18.3%) | 270.8(19.2%) |
| 24 |  |  |  |  |  |  |  |
| 25 | 643(58.7%) | 845(63.7%) | 889(65.5%) | 748(65.6%) | 990(72.2%) | 1057(74.0%) | 1011.8(71.7%) |
| 26 | 87(7.9%) | 116(8.7%) | 111(8.2%) | 89(7.8%) | 90(6.6%) | 121(8.5%) | 105.6(7.5%) |
| 27 | 366(33.4%) | 356(26.8%) | 357(26.3%) | 305(26.7%) | 291(21.2%) | 250(17.5%) | 292.8(20.8%) |
| 28 |  |  |  |  |  |  |  |
| 29 | 4\|**9**\|14 | 4\|**8**\|13 | 5\|**9**\|13 | 5\|**9**\|14 | 6\|**9**\|14 | 6\|**10**\|14 | 5\|**9**\|13 |
| 30 | 9\|**20**\|33 | 11\|**20**\|31 | 13\|**22**\|33 | 15\|**25**\|37 | 16\|**25**\|38 | 16\|**25**\|37 | 13\|**22**\|33 |
